# Economic evidence of alternative formulations and routes of administration for identical active pharmaceutical ingredient: a systematic review

**DOI:** 10.1186/s13561-026-00793-1

**Published:** 2026-05-20

**Authors:** Hanqiao Shao, Yunong Jiang, Mingye Zhao, Wenxi Tang

**Affiliations:** 1 School of International Pharmaceutical Business, China Pharmaceutical University, Nanjing, Jiangsu China; 2 Center for Pharmacoeconomics and Outcomes Research & Department of Public Affairs Management, School of International Pharmaceutical Business, China Pharmaceutical University, Nanjing, Jiangsu China

**Keywords:** Pharmacoeconomics, Drug administration routes, Dosage forms, Cost minimization analysis, Systematic review

## Abstract

**Background:**

Different formulations and routes of administration (e.g. oral, intravenous [IV], subcutaneous [SC]) of the same active pharmaceutical ingredient (API) may change value by altering administration burden, adherence, setting of care and resource use. This systematic review compared the economic impact of alternative formulations or routes of the same API and assessed reporting quality.

**Methods:**

PubMed and Embase were searched from inception to 31 January 2025 for full economic evaluations directly comparing dosage forms or administration routes of the same API in humans. Two reviewers independently screened records, extracted data, and appraised reporting quality using Consolidated Health Economic Evaluation Reporting Standards (CHEERS 2022). For cost minimization analyses (CMAs), costs were converted to 2024 US dollars (USD) and summarized as median annual total cost differences and relative changes. Mean CHEERS scores were compared between CMAs and non-CMAs.

**Results:**

Thirty-eight studies from 17 countries met criteria, spanning 11 ICD-11 categories and 10 route comparisons. CMA was most common (*n* = 20; 52.6%); all 18 non-CMA studies were model-based. Perspectives varied: healthcare system (42.1%), societal (36.8%), payer (15.8%), provider (15.8%). Industry funding was reported in 60.5%. CHEERS reporting was generally good; non-CMA studies scored higher than CMAs (mean 22.8 ± 0.9 vs. 20.9 ± 2.0; *p* < 0.001). Across indications, subcutaneous or oral administration versus intravenous were often associated with lower total costs, mainly through reductions in staff time, chair occupancy, consumables, travel, and productivity losses. In 18 CMAs with sufficient data, the median annual total cost difference was -$2,073 per patient (IQR -$3,594 to -$695), a -21.35% relative reduction (IQR − 30.96% to -5.24%). Subcutaneous versus intravenous comparisons showed a median annual difference of -$2,479 (IQR -$6,715 to -$1,943). Adherence was rarely modeled explicitly (10% of CMAs; 67% of non-CMAs), often based on assumptions; indirect and organizational costs were inconsistently captured.

**Conclusion:**

Alternative formulations and routes of the same API, particularly SC or oral instead of IV, often lower total treatment costs. However, frequent use of CMA, strong equivalence assumptions and incomplete costing limit robustness and support the need for more rigorous cost effectiveness analyses.

**Supplementary Information:**

The online version contains supplementary material available at 10.1186/s13561-026-00793-1.

## Introduction

Multiple formulations and routes of administration, including oral, intravenous, subcutaneous, transdermal, and inhaled delivery, are now available for many active pharmaceutical ingredients (APIs) [1, 2]. Although the active ingredient remains the same, formulation can influence effectiveness, safety, adherence, patient experience, care setting, and resource use [3–5]. These differences have direct implications for both patient outcomes and health‑system efficiency, for example by altering staff time, chair or bed occupancy, monitoring requirements, and the feasibility of home‑ or community‑based care [6, 7]. Accordingly, health technology assessment and methodological guidance has increasingly acknowledged that formulation and route may influence value, particularly when they affect adherence, workflow, or patient preferences [8–10].

Alongside first-in-class drug development, reformulation and route modification of established APIs have become an important part of pharmaceutical development [11]. By optimizing delivery modalities, these products leverage well-characterized pharmacology and existing clinical evidence, following more predictable regulatory pathways than entirely new molecular entities [12, 13]. This trend is reflected in the growing number of products approved through dedicated regulatory pathways, such as the US Food and Drug Administration’s 505(b)(2) pathway and China’s Class 2 route, where dosage-form and route changes represent the primary focus [14]. Evidence from regional markets, including over 70 improved new drugs approved in China between 2020 and 2025 alone [15], highlights how rapidly this sector is expanding through reformulations that enhance delivery while retaining the original active ingredient. Because the incremental benefits are often mediated through changes in resource use, time costs and care delivery rather than large differences in efficacy, rigorous economic evaluation is essential to clarify their net value for patients and health systems [16, 17].

This systematic review aimed to evaluate the economic impact of alternative formulations and routes of administration for identical active ingredients. Unlike previous reviews that have often focused on specific therapeutic areas, particularly oncology, or on selected route comparisons, this review was not restricted to a single disease area. We included only studies directly comparing alternative formulations or routes of the same active pharmaceutical ingredient and limited the analysis to full economic evaluations. Specifically, we assessed the clinical settings, modeling methodologies, and cost structures used in published studies, and examined the factors underlying reported differences in costs and outcomes.

## Methods

### Study design and eligibility

This systematic review was registered on PROSPERO (CRD42024513028) following the PRISMA (Preferred Reporting Items for Systematic Reviews and Meta-Analyses) guidelines [18, 19]. A detailed list of the search terms is provided in Supplementary Table 1. The search strategy and key terms were developed based on the PICOS (Population, Intervention, Comparison, Outcomes, Study design) framework. Studies were included if they met the following criteria:

1. Population: Human participants;
2. Intervention: Different dosage forms and/or administration routes of the same active pharmaceutical ingredient (API). The same API is defined strictly as the same active parent compound, excluding prodrugs, individual enantiomers or stereoisomers, and fixed-dose combinations or co-formulated products. Dosage form and administration route may vary between interventions;
3. Comparison: Direct, head‑to‑head comparisons between eligible dosage forms or administration routes of the same API;
4. Outcomes: Economic outcomes, including costs, cost differences, ICERs, cost per responder, or cost per life-year gained;
5. Study design: Pharmacoeconomic evaluations, including cost effectiveness analysis (CEA), cost utility analysis (CUA), cost minimization analysis (CMA), cost benefit analysis, and cost consequence analysis (CCA).

Studies were excluded based on the following criteria:

1. Non-primary research, including narrative reviews, systematic reviews, editorials, commentaries, letters, and study protocols;
2. Studies comparing different APIs, or studies not directly comparing alternative formulations or routes of the same API, were excluded;
3. Assessments of non-standard formulations (e.g., in-hospital preparations);
4. Non-English language publications.

### Search strategy and study selection

PubMed and Embase were searched from inception to 31 January 2025, using controlled vocabulary and free-text terms related to “drug administration routes”, “dosage forms”, and “economic evaluation” (full strategies in Supplementary Methods). The search strategy was developed in accordance with the recommendations of Mick Arber et al. [20] for identifying economic evaluations. Records were imported into Zotero (version 6.0.36). Two independent researchers (HS and YJ) independently screened titles and abstracts, with disagreements resolved through discussion or, when necessary, consultation with a third reviewer (MZ). Full text screening followed the same process.

### Data extraction and quality appraisal

Key study characteristics were extracted using a standardized data collection form, including first author, publication year, country, study design, perspective, and disease classification according to the World Health Organization International Classification of Diseases, 11th Revision (ICD-11) [21]. Intervention details, patient characteristics, cost and effectiveness results, and study conclusions were documented.

Study quality was independently assessed by two reviewers (HS and YJ) using the CHEERS 2022 checklist. Items were rated as “Yes” (fully addressed), “Partially” (partially addressed), or “No” (not addressed), with scores of 1, 0.5, and 0 points assigned, respectively. Disagreements were adjudicated by a third reviewer (WT). Based on the number of items fulfilled, studies were categorized as excellent (25–28 items), good (20–24), moderate (15–19), or low quality (≤ 14) [22, 23].

### Statistical analyses

For CMAs that reported costs by group and cost category, all costs were standardized to 2024 USD using the CCEMG-EPPI-Centre cost converter [24], first inflating values in the original currency to the target year using country-specific Gross Domestic Product deflators and then converting via purchasing power parity exchange rates. For each eligible study, the undiscounted absolute annual cost difference was calculated as intervention minus control, and the relative annual difference was calculated as the absolute difference divided by the control arm. Given the substantial heterogeneity across included studies in terms of settings, comparators, analytical methods, and reported outcomes, a meta-analysis was not considered appropriate. Therefore, we used a narrative and descriptive synthesis to summarize findings across all studies and reported annual differences within the cost minimization subset using medians and interquartile ranges. Differences in mean CHEERS scores between CMAs and non-CMA studies were compared using independent-samples t-tests. Statistical significance was assessed using two-sided tests with a significance level of *p* < 0.05, and p-values are reported descriptively.

## Results

### Study selection

**Fig. 1:**
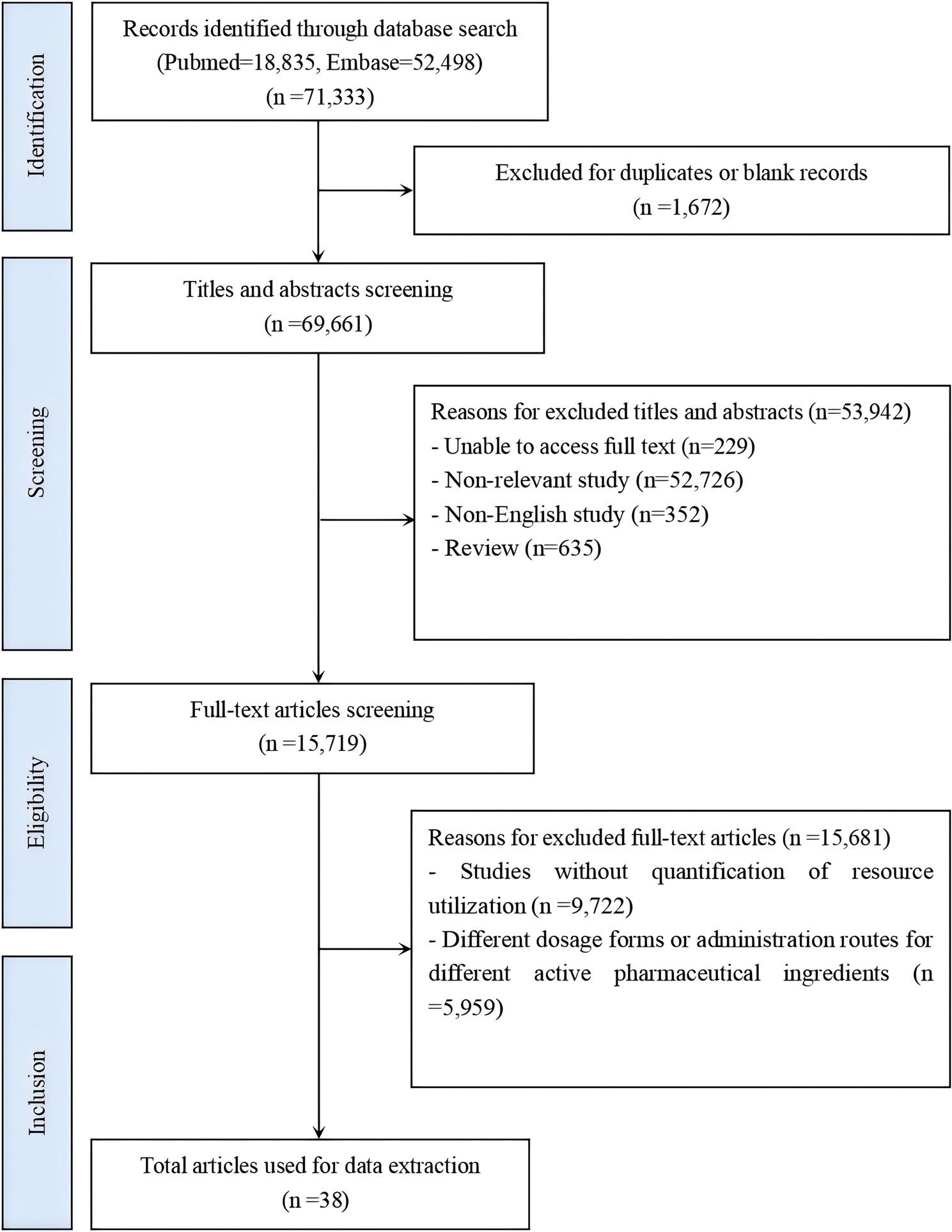
Flow diagram of literature search and study identification

A total of 71,333 records were identified from PubMed and Embase. After removal of 1,672 duplicates or blank records, 69,661 records were screened by title and abstract; 53,942 were excluded for irrelevance, lack of full‑text access, non‑English language, or being non‑original research. Full texts of 15,719 articles were assessed, of which 15,681 were excluded, most commonly because they did not compare alternative formulations or routes of the same API or did not report quantifiable resource use or economic outcomes. Ultimately, 38 studies [25–62] met all inclusion criteria and were included in the final analysis (Fig. 1).

### Study characteristics

**Fig. 2:**
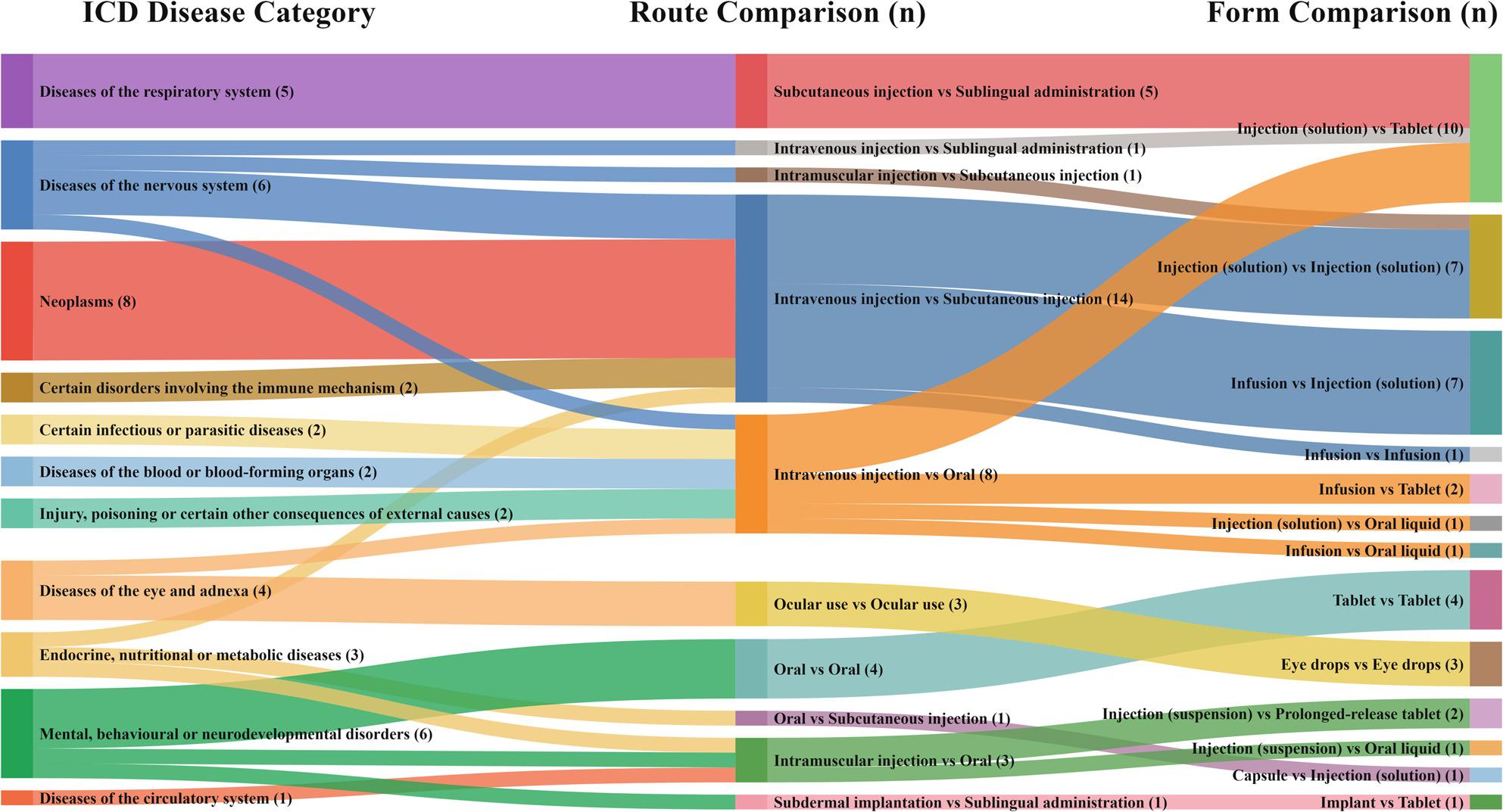
Comparisons of administration routes and dosage forms of the same active pharmaceutical ingredient by ICD disease category

The 38 included evaluations, published between 1996 and 2024, spanned 17 countries. Early work was mainly from Europe and North America; more recent studies also came from Asia and South America. No eligible studies originated from low-income countries. Sixteen evaluations involved biologics [25, 27–29, 33, 38–40, 42, 46, 50, 53, 54, 58], 20 involved small-molecule drugs [26, 30, 32, 34, 41, 43–45, 47–49, 52, 56, 57, 59–62] and five involved allergen extracts [31, 35–37, 55]. Eleven major disease categories by ICD-11 were represented, with the most frequent being neoplasms, mental and behavioral or neurodevelopmental disorders and diseases of the nervous system. Ten distinct route comparisons were identified, most often intravenous versus subcutaneous administration and intravenous versus oral administration. Thirteen dosage-form pairings were reported, most commonly injection solution versus tablet and injection solution versus injection solution. These patterns are illustrated in Fig. 2 and summarized in Table 1.

**Table 1:** Characteristics and methods of included studies

Category	*N* (%)
Overall included studies (*N* = 38)	
Types of analysis	
Cost minimization analysis	20 (52.63)
Cost effectiveness analysis	13 (34.21)
Cost utility analysis	4 (10.53)
Cost consequence analysis	1 (2.63)
Perspectives	
Societal	14 (36.84)
Healthcare system	16 (42.11)
Payer	6 (15.79)
Hospital/Provider	6 (15.79)
Funding type	
No fund	8 (21.05)
Non-profit fund	7 (18.42)
Profit fund	23 (60.53)
API category	
Biologic agents	16 (42.11)
Small‑molecule drugs	20 (52.63)
Allergen extracts	5 (13.16)
Non-Cost minimization analysis (*N* = 18)	
Model type	
Markov cohort	9 (50.00)
Decision tree	3 (16.67)
Microsimulation	4 (22.22)
Discrete event simulation	2 (11.11)
Cost identification	
Only DMCs	15 (83.33)
DMCs, ICs	2 (11.11)
DMCs, DNMCs, ICs	1 (5.56)
Clinical data source	
Randomized controlled trials	11 (61.11)
Observational studies	7 (38.89)
Adherence consideration	
Randomized controlled trials	2 (11.11)
Real-world data	5 (27.78)
Expert judgement	3 (16.67)
Assumptions	2 (11.11)
Not considered	6 (33.33)
Cost minimization analysis (*N* = 20)	
Clinical data source	
Randomized controlled trials	14 (70.00)
Observational studies	2 (10.00)
Systematic reviews	2 (10.00)
Equivalence assumption	2 (10.00)
Cost identification	
Only DMCs	10 (50.00)
DMCs, ICs	7 (35.00)
DMCs, DNMCs, ICs	3 (15.00)
Adherence consideration	
Assumption-based	2 (10.00)
Not considered	18 (90.00)

Some studies adopted more than one evaluation perspective, and a single study may assess more than one active pharmaceutical ingredient

*DMCs *Direct medical costs, *DNMCs *Direct non-medical costs, *ICs *Indirect costs

### Methodological approaches

CMA was the predominant analytic framework (*n* = 20; 52.6%) [27–29, 31, 33–37, 42, 46, 49–51, 53–55, 58, 60, 61], followed by CEA (*n* = 13; 34.2%) [25, 26, 30, 32, 39–41, 47, 52, 56, 57, 59, 62], CUA (*n* = 4; 10.5%) [43–45, 48], and one cost consequence analysis [38]. Perspectives varied: healthcare system (*n* = 16; 42.1%) [27–29, 31, 34, 40, 41, 43–45, 47, 50, 54–56, 61], societal (*n* = 14; 36.8%) [27–29, 31, 32, 35, 36, 42, 48, 52–54, 57, 59], payer (*n* = 6; 15.8%) [25, 30, 37, 39, 60, 62], and hospital/provider (*n* = 6; 15.8%) [26, 33, 46, 49, 51, 58]. Over half of studies (*n* = 23; 60.5%) reported industry funding [27–37, 39, 42–45, 47, 49–51, 54, 55, 59]; eight reported no external funding [38, 46, 48, 53, 58, 60–62] and seven were supported by non-profit organizations [25, 26, 40, 41, 52, 56, 57].

The 20 CMAs [27–29, 31, 33–37, 42, 46, 49–51, 53–55, 58, 60, 61] all relied on an assumption of equal efficacy and safety between comparator formulations or routes of the same API, supported by head-to-head randomized controlled trials (RCTs) in 14 CMAs (70.0%) [27–29, 31, 33–35, 37, 46, 50, 51, 53, 54, 58], observational comparative data in 2 (10.0%) [49, 60], indirect comparisons or broader evidence syntheses [55, 61] in 2 (10.0%), and other sources in 2 (10.0%) [36, 42]. Regarding cost components, 10 CMAs (50.0%) [33, 34, 46, 49–51, 54, 58, 60, 61] considered only direct medical costs, 7 (35.0%) [27–29, 35–37, 55] included both direct medical and indirect costs, and 3 (15.0%) [31, 42, 53] comprehensively incorporated direct medical, direct non-medical, and indirect costs. Treatment adherence was rarely addressed: only 2 CMAs (10.0%) [55, 61] explicitly modelled differential adherence, both using investigator-driven assumptions rather than empirical adherence data, whereas the remaining 18 CMAs (90.0%) [27–29, 31, 33–37, 42, 46, 49–51, 53, 54, 58, 60] did not account for patient adherence.

All 18 non-CMA evaluations (13 CEAs [25, 26, 30, 32, 39–41, 47, 52, 56, 57, 59, 62], 4 CUAs [43–45, 48], and 1 CCA [38]) were model-based. Cohort-based Markov or other state transition models were used in 9 studies [26, 32, 43, 44, 48, 52, 59, 62], decision trees in 3 [38, 56, 57], patient-level microsimulation or Monte Carlo models in 4 [25, 30, 40, 41], and discrete-event simulation in 2 [39, 47]. Time horizons ranged from 1 year to lifetime. Regarding costs, 15 non-CMA studies included only direct medical costs [25, 26, 30, 38–41, 43–45, 47, 52, 56, 59, 62], 2 [32, 57] incorporated both direct medical and indirect costs, and only 1 [48] evaluation comprehensively considered direct medical, direct non-medical, and indirect costs. For clinical data sources, 11 [25, 30, 32, 38, 39, 43–45, 56, 59, 62] studies were primarily informed by RCTs, while 7 [26, 40, 41, 47, 48, 52, 57] relied mainly on observational data. Twelve non-CMA evaluations explicitly incorporated adherence: 2 based on adherence data from RCTs [32, 39], 5 using real-world adherence data [25, 30, 47, 57, 62], 3 informed by expert opinion [43–45], and 2 relying on adherence assumptions [41, 52]. The remaining 6 studies did not consider patient adherence [26, 38, 40, 48, 56, 59]. Detailed characteristics of the included studies are provided in Table 1 and Supplementary Table 2.

### Economic outcomes

In most included studies, alternative formulations or routes of administration for the same active ingredient were associated with lower costs. Clinical outcomes were generally reported as similar between comparators, although some studies also described differences in patient-reported or treatment-related outcomes.

#### Findings from non‑CMA evaluations

Across mental and behavioral disorders, model-based evaluations generally found that adherence-enhancing formulations (long-acting injectables, orally disintegrating tablets, subdermal implants) reduced relapses and hospitalizations versus standard oral tablets, but often at substantially higher drug costs and ICERs above conventional thresholds [62]. In opioid use disorder a US societal-perspective model showed subdermal buprenorphine implants increased 12-month abstinence from 54% to 75% and retention from 58% to 78%, while lowering total costs by about $4,400 and gaining 0.031 QALYs per patient [32]. In nervous system diseases, oral phenytoin loading in the emergency department, despite a longer time to safe discharge (6.4 vs. 1.7 h), was far cheaper ($2.83 vs. $176.79) and remained the most cost-effective option, whereas in amyotrophic lateral sclerosis sublingual edaravone added only 0.034 QALYs at an extra ¥12,670 over 20 years when both formulations were home-based, but became dominant (-¥26,700; +0.234 QALYs) when compared with hospital-based IV infusion [48]. In hematologic and nutritional conditions, intravenous iron for pregnant women with moderate-to-severe iron-deficiency anemia achieved extra QALYs at acceptable incremental costs, while in pediatric intestinal failure daily enteral iron avoided 0.3 transfusions per patient at about $34 per transfusion avoided, whereas monthly parenteral iron avoided 0.5 transfusions but at an additional $6,600 per transfusion [41]. In ophthalmology, latanoprost cationic emulsion dominated standard latanoprost via improved adherence and downstream cost offsets [43–45]. In endocrine and metabolic disease [25], oral semaglutide and continuous SC insulin infusion improved glycemic control and quality of life at incremental ratios within or near typical thresholds despite higher acquisition costs (Supplementary Tables 3–4).

#### Findings from CMAs

CMAs consistently showed that alternative routes of administration, particularly subcutaneous or oral administration compared with intravenous delivery, were associated with lower total costs while maintaining assumed equivalence in clinical outcomes. Switching from intravenous to subcutaneous trastuzumab or rituximab in breast cancer and non-Hodgkin lymphoma reduced per-administration costs (trastuzumab €1,781 IV vs. €1,701 SC; rituximab €2,116 IV vs. €1,941 SC) and, in an Italian day-hospital CMA, lowered the annual drug-related budget by about €61,000 under current use and by a further €174,000 in a scenario of near-universal SC adoption [28]. These savings were primarily driven by shorter administration time, reduced demand for infusion chairs, avoidance of central venous access, and lower hospital overheads. Similar findings were reported for subcutaneous versus intravenous immunoglobulin in primary immunodeficiency [27, 50] and chronic inflammatory demyelinating polyneuropathy [42, 53], as well as for sublingual versus subcutaneous immunotherapy in allergic respiratory disease, where fewer clinic visits, reduced travel, and lower productivity losses contributed substantially to overall cost reductions (Supplementary Table 5).

### Quality appraisal

**Fig. 3:**
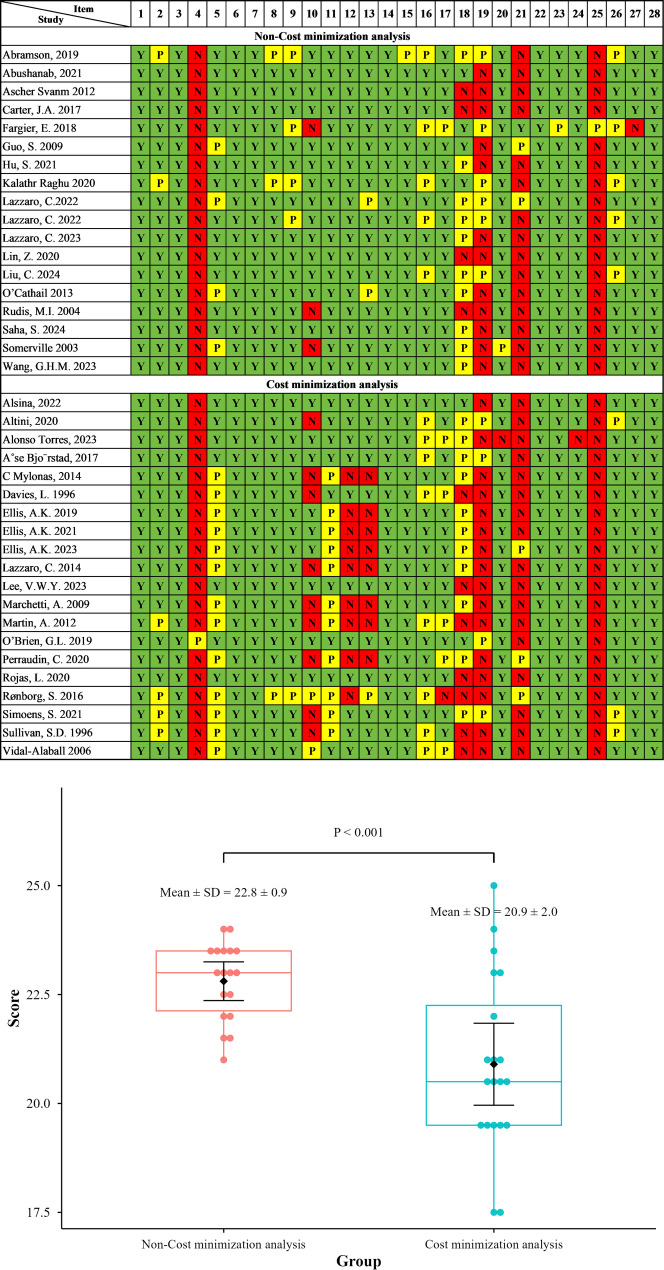
CHEERS 2022 reporting quality (**A**) Study-level CHEERS 2022 checklist scores; (**B**) Comparison of CHEERS 2022 reporting scores between cost minimization and non-cost minimization analyses. Note: Each study was assessed against every item in the checklist. Y indicates “Yes”, meaning the item is fully addressed; N indicates “No”, meaning the item is not addressed; P indicates “Partially”, meaning the item is partially addressed. For scoring purposes, Y was assigned 1 point, P 0.5 points, and N 0 points. Items correspond to CHEERS 2022 reporting standards: 1. Title; 2. Abstract; 3. Background and objectives; 4. Health economic analysis plan; 5. Study population; 6. Setting and location; 7. Comparators; 8. Perspective; 9. Time horizon; 10. Discount rate; 11. Selection of outcomes; 12. Measurement of outcomes; 13. Valuation of outcomes; 14. Measurement and valuation of resources and costs; 15. Currency, price date, and conversion; 16. Rationale and description of model; 17. Analytics and assumptions; 18. Characterizing heterogeneity; 19. Characterizing distributional effects; 20. Characterizing uncertainty; 21. Approach to engagement with patients and others affected by the study; 22. Study parameters; 23. Summary of main results; 24. Effect of uncertainty; 25. Effect of engagement with patients and others affected by the study; 26. Study findings, limitations, generalizability, and current knowledge; 27. Source of funding; 28. Conflicts of interest

CHEERS 2022 appraisal rated 30/38 studies (78.9%) as good or excellent; 8 were moderate; none were low. Non‑CMA evaluations had higher mean CHEERS scores than CMAs (22.8 ± 0.9 vs. 20.9 ± 2.0; *p* < 0.001), reflecting more complete reporting of perspective, methods, assumptions, and uncertainty. Item‑level performance and study‑level scores are shown in Fig. 3.

Despite the generally favorable performance, several reporting domains consistently exhibited weaknesses across studies. Most evaluations provided limited or no information regarding the health economic analysis plan, the approach to engaging patients or other stakeholders, and the effects of such engagement on study design or interpretation. These omissions suggest insufficient transparency in pre‑specified analytical procedures and minimal incorporation of patient or stakeholder input into economic evaluations.

### Quantified annual cost differences in CMAs

**Fig. 4:**
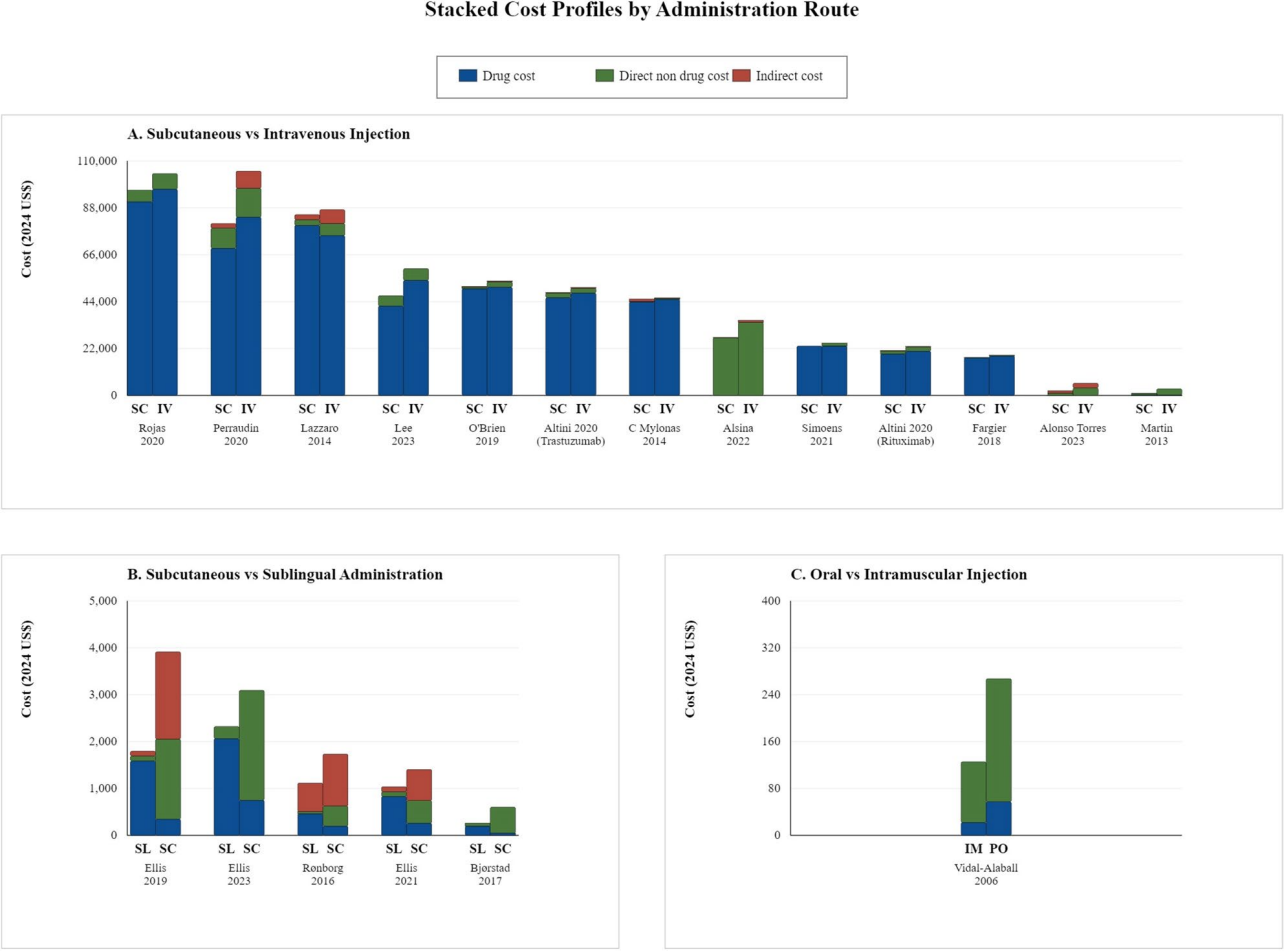
Annual treatment costs in cost minimization analyses. Note: IM, Intramuscular injection; IV, Intravenous; PO, Oral; SC, Subcutaneous; SL, Sublingual administration

Eighteen CMAs reported sufficient data for cost standardization and comparison. After converting to 2024 USD, the median absolute annual total cost difference (intervention minus control) was -$2,073.33 per patient (IQR -$3,594.35 to -$695.21), corresponding to a median relative reduction of -21.35% (IQR − 30.96% to -5.24%) (Supplementary Table 6; Fig. 4). For route‑of‑administration subgroups: SC versus IV was associated with a median annual difference of -$2,479.37 (IQR -$6,715.08 to -$1,943.21) and a median relative reduction of -7.87% (IQR − 23.20% to -5.04%); sublingual versus subcutaneous showed -$2,201.77 (IQR -$3,604.33 to -$850.88) and − 22.06% (IQR −33.30% to -5.66%), respectively.

### Impact of formulation and administration route on costs and outcomes

Several consistent sources explained the economic variations observed across studies. Analyses that compared subcutaneous or oral administration with intravenous infusion, and that explicitly incorporated staff time [29, 42, 58], chair occupancy [54], consumable or patient time costs [28, 34, 42, 50, 51, 54, 55, 58], consistently identified delivery related efficiencies that often offset similar or even higher drug acquisition prices. Evaluations conducted from societal or provider perspectives captured a larger proportion of these efficiencies than those using restricted payer perspectives. A smaller number of evaluations incorporated adherence into the analysis. In these studies, formulations associated with lower treatment burden or simpler administration were sometimes linked to better effectiveness or quality-of-life outcomes, although the methods used to define and model adherence varied across studies. Industry funding was common, but no clear difference in the direction of findings was apparent at the descriptive level. Even so, the possibility of sponsor-related bias cannot be excluded. Relevant cost components and study features are summarized in Supplementary Table 7.

## Discussion

### Main findings

This systematic review found that many included studies reported lower costs for alternative formulations or routes of the same active ingredient, particularly when administration became less resource intensive. Evidence for differences in clinical or patient-reported outcomes was more limited and less consistent. In most studies, cost differences were mainly related to administration time, healthcare staff input, treatment capacity, travel, and other service-delivery factors rather than differences in the pharmacological effect of the active ingredient. Shorter administration and monitoring time reduced use of infusion chairs and central access, fewer inpatient days and procedures and lower patient and caregiver time costs featured prominently as sources of savings. In selected contexts, improvements in adherence translated into fewer relapses or complications and better quality of life.

More than half of the included studies used CMA, which affects how the findings should be interpreted. CMA may be reasonable when comparable efficacy and safety have been established. In practice, however, changes in formulation or route may still affect absorption, onset, treatment burden, administration-related adverse events, or acceptability, and these differences may matter in routine care. Regimens also differ in their resilience to missed or delayed doses; small changes in effective half-life relative to dosing interval can influence how forgiving a regimen is to imperfect adherence [63, 64].

Current health technology assessment guidance suggests that cost-minimization analysis is appropriate only when comparators are demonstrably equivalent in clinically relevant outcomes. The National Institute for Health and Care Excellence notes that differences in costs related to health outcomes, such as adverse events, may indicate that overall health benefits are not truly comparable and therefore require justification [8]. The Pharmaceutical Benefits Advisory Committee similarly requires evidence that the proposed intervention is non-inferior or superior in efficacy and equivalent or better in safety [9]. The Canadian Agency for Drugs and Technologies in Health takes a similar position, limiting the use of cost-minimization analysis to situations in which equivalence has been adequately established [10]. In these settings, CEA or CUA may be more informative than cost-minimization analysis [65].

Treatment of adherence was uneven. Only a minority of studies, mainly in schizophrenia [30, 47, 62], diabetes [25, 40] and glaucoma [43–45], explicitly modelled formulation-specific adherence and linked it to outcomes such as relapse, hospitalization or long-term complications, despite the fact that many formulation innovations are designed to address adherence barriers. Because many formulation innovations are intended to reduce treatment burden or improve persistence, future models should incorporate adherence differences when clinically plausible, justify assumptions transparently, and test their influence through scenario and probabilistic sensitivity analyses [66–68].

Broader value components related to time, workflow and patient experience were incompletely captured. The savings observed were primarily delivery efficiencies rather than drug price differentials, yet many evaluations labelled as societal omitted patient and caregiver time [69], productivity losses [70] or organizational efficiencies [71]. Patient-centered outcomes such as convenience, treatment burden and satisfaction were inconsistently measured or integrated into incremental analyses [72]. Future evaluations would benefit from more consistent and transparent costing, with separate reporting of drug acquisition, consumables, staff time, treatment capacity, monitoring, and patient or caregiver time where relevant [73]. Approaches that combine time-and-motion or workflow studies with model-based analyses may help clarify how changes in route or setting affect resource use and capacity constraints [74].

### Limitations

The evidence base underlying this review has several important limitations. First, the included studies were heterogeneous in terms of indications, healthcare settings, perspectives, time horizons, model structures, cost components, and the granularity of resource-use measurement. Accordingly, the synthesis of cost findings, especially the summary of annual cost differences across CMAs and route comparisons, should be interpreted as descriptive rather than quantitative pooling. Reported “typical” savings may mask substantial variation related to local practice, wage levels, capacity constraints, and implementation models.

Second, more than half of the included studies were industry funded, and most were conducted in high-income or upper-middle-income settings, which may limit the generalizability of the findings. Cost patterns observed in these settings may not transfer directly to low- and middle-income countries, where care delivery, infrastructure, patient travel burden, and out-of-pocket spending differ substantially. Third, relatively few studies conducted extensive scenario or probabilistic sensitivity analyses around key structural assumptions, including clinical equivalence, adherence, and time valuation, which adds further uncertainty to the interpretation of reported cost and cost-effectiveness patterns.

### Future research directions

Several gaps in the current evidence base should be addressed in future studies. Future studies should report adherence, healthcare use, and patient-reported outcomes more directly under routine care conditions [75]. Economic evaluations should also examine whether differences in convenience, treatment burden, or adherence are likely to affect outcomes and costs [64, 76]. In addition, costing should be reported more transparently, with clearer separation of staff time, treatment capacity, travel, and other non-drug costs [77]. Evidence remains limited in low- and middle-income settings and in systems moving toward ambulatory or home-based care.

## Conclusions

In this systematic review, alternative formulations or routes of the same active ingredient were often associated with lower costs, especially when less resource-intensive administration replaced intravenous delivery. Reported differences were mainly related to treatment delivery, staff time, and other service-use components. However, the evidence base is limited by the widespread use of cost minimization analyses, frequent assumptions of clinical equivalence, and incomplete capture of indirect and organizational costs. Future evaluations should move beyond simple cost comparisons when formulation or route changes are likely to affect adherence, patient experience, or downstream outcomes.

## Supplementary Information

Supplementary Material 1.

## Data Availability

This study relied solely on data that are publicly accessible in databases such as PubMed and EMBASE. No confidential information or data subject to data use agreements were involved.

## Abbreviations

API: Active pharmaceutical ingredient
CCA: Cost consequence analysis
CEA: Cost effectiveness analysis
CHEERS: Consolidated Health Economic Evaluation Reporting Standards
CMA: Cost minimization analysis
CUA: Cost utility analysis
DMCs: Direct medical costs
DNMCs: Direct non-medical costs
ICD-11: International Classification of Diseases, 11th revision
ICER: Incremental cost-effectiveness ratio
ICs: Indirect costs
IM: Intramuscular injection
IQR: Interquartile range
IV: Intravenous
PO: Oral
PPP: Purchasing power parity
QALYs: Quality-adjusted life years
RCTs: Randomized controlled trials
SC: Subcutaneous
SL: Sublingual administration
USD: US dollars
