# Supplementary material for: Economic evidence of alternative formulations and routes of administration for identical active pharmaceutical ingredient: a systematic review
Source: Health Econ Rev. 2026 May 20;16:86. doi: 10.1186/s13561-026-00793-1 (PMC13366963; doi:10.1186/s13561-026-00793-1)
Supplement: Supplementary file 1 — Supplementary Material 1. [file 13561_2026_793_MOESM1_ESM.docx]

**Supplementary Content**

[Table 1 PRISMA Guidelines for Systematic Reviews 2](#_Toc9746)

[Methods Search Strategies 6](#_Toc27763)

[Table 2 Summaries of Main Characters of Included Studies 12](#_Toc31965)

[Table 3 Methodological Features of Included Cost-Effectiveness and Cost-Utility Analyses 29](#_Toc20074)

[Table 4 Key Characteristics and Main Findings of Included Cost-Effectiveness and Cost-Utility Analyses 35](#_Toc2326)

[Table 5 Key Characteristics of Included Cost-Minimization Analyses Studies 41](#_Toc25612)

[Table 6 Annual Costs and Cost Differences in Included Cost-Minimization Analyses Studies 46](#_Toc10088)

[Table 7 Formulation and Route of Administration Influence Economic Evaluation Results 50](#_Toc16652)

[Reference 57](#_Toc26535)

**Table 1 PRISMA Guidelines for Systematic Reviews**

| **Section and Topic** | **Item #** | **Checklist item** | **Location where item is reported** |
| --- | --- | --- | --- |
| **TITLE** | | |  |
| Title | 1 | Identify the report as a systematic review. | Title |
| **ABSTRACT** | | |  |
| Abstract | 2 | See the PRISMA 2020 for Abstracts checklist. | Abstract |
| **INTRODUCTION** | | |  |
| Rationale | 3 | Describe the rationale for the review in the context of existing knowledge. | Introduction paragraphs1-3 |
| Objectives | 4 | Provide an explicit statement of the objective(s) or question(s) the review addresses. | Introduction paragraph 3 |
| **METHODS** | | |  |
| Eligibility criteria | 5 | Specify the inclusion and exclusion criteria for the review and how studies were grouped for the syntheses. | Methods (2.1 Study Design and Eligibility；2.4 Statistical Analyses) |
| Information sources | 6 | Specify all databases, registers, websites, organisations, reference lists and other sources searched or consulted to identify studies. Specify the date when each source was last searched or consulted. | Methods (2.2 Searching Strategy and Study Selection) |
| Search strategy | 7 | Present the full search strategies for all databases, registers and websites, including any filters and limits used. | Methods (2.2 Searching Strategy and Study Selection) |
| Selection process | 8 | Specify the methods used to decide whether a study met the inclusion criteria of the review, including how many reviewers screened each record and each report retrieved, whether they worked independently, and if applicable, details of automation tools used in the process. | Methods (2.2 Searching Strategy and Study Selection) |
| Data collection process | 9 | Specify the methods used to collect data from reports, including how many reviewers collected data from each report, whether they worked independently, any processes for obtaining or confirming data from study investigators, and if applicable, details of automation tools used in the process. | Methods (2.3 Data Extraction and Quality Appraisal) |
| Data items | 10a | List and define all outcomes for which data were sought. Specify whether all results that were compatible with each outcome domain in each study were sought (e.g. for all measures, time points, analyses), and if not, the methods used to decide which results to collect. | Methods (2.3 Data Extraction and Quality Appraisal；2.4 Statistical Analyses) |
|  | 10b | List and define all other variables for which data were sought (e.g. participant and intervention characteristics, funding sources). Describe any assumptions made about any missing or unclear information. | Methods (2.3 Data Extraction and Quality Appraisal) |
| Study risk of bias assessment | 11 | Specify the methods used to assess risk of bias in the included studies, including details of the tool(s) used, how many reviewers assessed each study and whether they worked independently, and if applicable, details of automation tools used in the process. | Methods (2.3 Data Extraction and Quality Appraisal) |
| Effect measures | 12 | Specify for each outcome the effect measure(s) (e.g. risk ratio, mean difference) used in the synthesis or presentation of results. | Methods (2.4 Statistical Analyses) |
| Synthesis methods | 13a | Describe the processes used to decide which studies were eligible for each synthesis (e.g. tabulating the study intervention characteristics and comparing against the planned groups for each synthesis (item #5)). | Methods (2.4 Statistical Analyses) |
|  | 13b | Describe any methods required to prepare the data for presentation or synthesis, such as handling of missing summary statistics, or data conversions. | Methods (2.4 Statistical Analyses) |
|  | 13c | Describe any methods used to tabulate or visually display results of individual studies and syntheses. | Methods (2.4 Statistical Analyses); Results 3.2-3.3; Table 1; Supplementary Tables 2-7; Figures 2-4 |
|  | 13d | Describe any methods used to synthesize results and provide a rationale for the choice(s). If meta-analysis was performed, describe the model(s), method(s) to identify the presence and extent of statistical heterogeneity, and software package(s) used. | Methods (2.4 Statistical Analyses) |
|  | 13e | Describe any methods used to explore possible causes of heterogeneity among study results (e.g. subgroup analysis, meta-regression). | N/A |
|  | 13f | Describe any sensitivity analyses conducted to assess robustness of the synthesized results. | N/A |
| Reporting bias assessment | 14 | Describe any methods used to assess risk of bias due to missing results in a synthesis (arising from reporting biases). | Methods (2.3 Data Extraction and Quality Appraisal) |
| Certainty assessment | 15 | Describe any methods used to assess certainty (or confidence) in the body of evidence for an outcome. | Methods (2.3 Data Extraction and Quality Appraisal) |
| **RESULTS** | | |  |
| Study selection | 16a | Describe the results of the search and selection process, from the number of records identified in the search to the number of studies included in the review, ideally using a flow diagram. | Results 3.1 Study Selection; Figure 1 |
|  | 16b | Cite studies that might appear to meet the inclusion criteria, but which were excluded, and explain why they were excluded. | Results (3.1 Study Selection) |
| Study characteristics | 17 | Cite each included study and present its characteristics. | Results 3.2 Study Characteristics; Table 1；Supplementary Table 2 |
| Risk of bias in studies | 18 | Present assessments of risk of bias for each included study. | Results 3.5 Quality Appraisal |
| Results of individual studies | 19 | For all outcomes, present, for each study: (a) summary statistics for each group (where appropriate) and (b) an effect estimate and its precision (e.g. confidence/credible interval), ideally using structured tables or plots. | Results 3.3-3.4; Supplementary Tables 3-6； Figure 4 |
| Results of syntheses | 20a | For each synthesis, briefly summarize the characteristics and risk of bias among contributing studies. | Results (3.3 Methodological Approaches；3.5 Quality Appraisal) |
|  | 20b | Present results of all statistical syntheses conducted. If meta-analysis was done, present for each the summary estimate and its precision (e.g. confidence/credible interval) and measures of statistical heterogeneity. If comparing groups, describe the direction of the effect. | Results (3.5 Quality Appraisal; 3.6 Quantified Annual Cost Differences in CMAs) |
|  | 20c | Present results of all investigations of possible causes of heterogeneity among study results. | Results (3.7 Impact of Formulation and Administration Route on Costs and Outcomes) |
|  | 20d | Present results of all sensitivity analyses conducted to assess the robustness of the synthesized results. | N/A |
| Reporting biases | 21 | Present assessments of risk of bias due to missing results (arising from reporting biases) for each synthesis assessed. | Results (3.5 Quality Appraisal) |
| Certainty of evidence | 22 | Present assessments of certainty (or confidence) in the body of evidence for each outcome assessed. | Results (3.5 Quality Appraisal) |
| **DISCUSSION** | | |  |
| Discussion | 23a | Provide a general interpretation of the results in the context of other evidence. | Discussion (4.1 Main Findings) |
|  | 23b | Discuss any limitations of the evidence included in the review. | Discussion (4.2 Limitations) |
|  | 23c | Discuss any limitations of the review processes used. | Discussion (4.2 Limitations) |
|  | 23d | Discuss implications of the results for practice, policy, and future research. | Discussion (4.3 Future Research Directions) |
| **OTHER INFORMATION** | | |  |
| Registration and protocol | 24a | Provide registration information for the review, including register name and registration number, or state that the review was not registered. | Methods (2.1 Study Design and Eligibility) |
|  | 24b | Indicate where the review protocol can be accessed, or state that a protocol was not prepared. | Methods (2.1 Study Design and Eligibility) |
|  | 24c | Describe and explain any amendments to information provided at registration or in the protocol. | N/A |
| Support | 25 | Describe sources of financial or non-financial support for the review, and the role of the funders or sponsors in the review. | Declaration |
| Competing interests | 26 | Declare any competing interests of review authors. | Declaration |
| Availability of data, code and other materials | 27 | Report which of the following are publicly available and where they can be found: template data collection forms; data extracted from included studies; data used for all analyses; analytic code; any other materials used in the review. | Declaration |

Note: “N/A” indicates not applicable.

# Methods Search Strategies

The PubMed search yielded 18,835 records using the strategy.

| **Search ID** | **Search Details** |
| --- | --- |
| #1 | (((Capsules [Mesh]) OR (Capsule [Title/Abstract]) OR (Microcapsules [Title/Abstract]) OR (Microcapsule [Title/Abstract]) OR (Nasal Sprays [Mesh]) OR (Sprays, Nasal [Title/Abstract]) OR (Nasal Mist [Title/Abstract]) OR (Mist, Nasal [Title/Abstract]) OR (Nasal Aerosol [Title/Abstract]) OR (Aerosol, Nasal [Title/Abstract]) OR (Nasal Spray [Title/Abstract]) OR (Spray, Nasal [Title/Abstract]) OR (Oral Sprays [Mesh]) OR (Sprays, Oral [Title/Abstract]) OR (Oral Spray [Title/Abstract]) OR (Spray, Oral [Title/Abstract]) OR (Emulsions [Mesh]) OR (Emulsion [Title/Abstract]) OR (Fat Emulsions, Intravenous [Mesh]) OR (Emulsions, Intravenous Fat [Title/Abstract]) OR (Intravenous Fat Emulsions [Title/Abstract]) OR (Lipid Emulsions, Intravenous [Title/Abstract]) OR (Emulsions, Intravenous Lipid [Title/Abstract]) OR (Intravenous Lipid Emulsions [Title/Abstract]) OR (Intravenous Lipid Emulsion [Title/Abstract]) OR (Emulsion, Intravenous Lipid [Title/Abstract]) OR (Lipid Emulsion, Intravenous [Title/Abstract]) OR (Intravenous Fat Emulsion [Title/Abstract]) OR (Emulsion, Intravenous Fat [Title/Abstract]) OR (Fat Emulsion, Intravenous [Title/Abstract]) OR (Liniments [Mesh]) OR (Ointments [Title/Abstract]) OR (Ointment [Title/Abstract]) OR (Unguents [Title/Abstract]) OR (Salve [Title/Abstract]) OR (Salves [Title/Abstract]) OR (Unguent [Title/Abstract]) OR (Pastes [Title/Abstract]) OR (Paste [Title/Abstract]) OR (Skin Ointment [Title/Abstract]) OR (Ointment, Skin [Title/Abstract]) OR (Powders [Mesh]) OR (Powder [Title/Abstract]) OR (Suppositories [Mesh]) OR (Vaginal Suppositories [Title/Abstract]) OR (Suppositories, Vaginal [Title/Abstract]) OR (Vaginal Suppository [Title/Abstract]) OR (Suppository, Vaginal [Title/Abstract]) OR (Rectal Suppositories [Title/Abstract]) OR (Suppositories, Rectal [Title/Abstract]) OR (Tablets [Mesh]) OR (Tablet [Title/Abstract]) OR (Vaginal Creams, Foams, and Jellies [Mesh]) OR (Vaginal Creams, Foams and Jellies [Title/Abstract]) OR (Vaginal Tablet [Title/Abstract]) OR (Tablet, Vaginal [Title/Abstract]) OR (Tablets, Vaginal [Title/Abstract]) OR (Vaginal Tablets [Title/Abstract]) OR (Vaginal Jelly [Title/Abstract]) OR (Jellies, Vaginal [Title/Abstract]) OR (Jelly, Vaginal [Title/Abstract]) OR (Vaginal Jellies [Title/Abstract]) OR (Vaginal Gel [Title/Abstract]) OR (Gel, Vaginal [Title/Abstract]) OR (Gels, Vaginal [Title/Abstract]) OR (Vaginal Gels [Title/Abstract]) OR (Vaginal Creams [Title/Abstract]) OR (Cream, Vaginal [Title/Abstract]) OR (Creams, Vaginal [Title/Abstract]) OR (Vaginal Cream [Title/Abstract]) OR (Vaginal Foams [Title/Abstract]) OR (Foam, Vaginal [Title/Abstract]) OR (Foams, Vaginal [Title/Abstract]) OR (Vaginal Foam [Title/Abstract])) OR ((Administration, Inhalation [Mesh]) OR (Administration, Intravenous [Mesh]) OR (Infusions, Intravenous [Mesh]) OR (Injections, Intravenous [Mesh]) OR (Administration, Oral [Mesh]) OR (Administration, Buccal [Mesh]) OR (Administration, Sublingual [Mesh]) OR (Administration, Topical [Mesh]) OR (Administration, Buccal [Mesh]) OR (Administration, Cutaneous [Mesh]) OR (Administration, Intravaginal [Mesh]) OR (Administration, Intravesical [Mesh]) OR (Administration, Mucosal [Mesh]) OR (Administration, Ophthalmic [Mesh]) OR (Chemotherapy, Cancer, Regional Perfusion [Mesh]) OR (Infusions, Parenteral [Mesh]) OR (Infusions, Intra-Arterial [Mesh]) OR (Infusions, Intralesional [Mesh]) OR (Infusions, Intraosseous [Mesh]) OR (Infusions, Intravenous [Mesh]) OR (Infusions, Intraventricular [Mesh]) OR (Infusions, Spinal [Mesh]) OR (Infusions, Subcutaneous [Mesh]) OR(Injections [Mesh]) OR (Injection, Intratympanic [Mesh]) OR (Injections, Intra-Arterial [Mesh]) OR (Injections, Intra-Articular [Mesh]) OR (Injections, Intralesional [Mesh]) OR (Injections, Intralymphatic [Mesh]) OR (Injections, Intramuscular [Mesh]) OR (Injections, Intraocular [Mesh]) OR (Injections, Intraperitoneal [Mesh]) OR (Injections, Intravenous [Mesh]) OR (Injections, Intraventricular [Mesh]) OR (Injections, Spinal [Mesh]) OR (Injections, Subcutaneous [Mesh]) OR (Microinjections [Mesh]))) |
| #2 | (((((Analysis, Cost-Effectiveness [Title/Abstract]) OR (Cost Effectiveness Analysis [Title/Abstract]) OR (Cost Effectiveness [Title/Abstract]) OR (Effectiveness, Cost [Title/Abstract]) OR (Cost Effectiveness Ratio [Title/Abstract]) OR (Cost Effectiveness Ratios [Title/Abstract]) OR (Effectiveness Ratio, Cost [Title/Abstract]) OR (Ratio, Cost Effectiveness [Title/Abstract]) OR (Cost-Effectiveness Analysis [Mesh])) OR ((Cost-Benefit Analysis [Mesh]) OR (Analysis, Cost-Benefit [Title/Abstract]) OR (Cost-Benefit Analyses [Title/Abstract]) OR (Cost Benefit Analysis [Title/Abstract]) OR (Analyses, Cost Benefit [Title/Abstract]) OR (Analysis, Cost Benefit [Title/Abstract]) OR (Cost Benefit Analyses [Title/Abstract]) OR (Cost-Utility Analysis [Title/Abstract]) OR (Analysis, Cost-Utility [Title/Abstract]) OR (Cost Utility Analysis [Title/Abstract]) OR (Cost-Utility Analyses [Title/Abstract]) OR (Cost Benefit [Title/Abstract]) OR (Costs and Benefits [Title/Abstract]) OR (Benefits and Costs [Title/Abstract]) OR (Cost and Benefit [Title/Abstract]) OR (Benefit and Cost [Title/Abstract]) OR (Marginal Analysis [Title/Abstract]) OR (Analysis, Marginal [Title/Abstract]) OR (Marginal Analyses [Title/Abstract]) OR (Cost-Benefit Data [Title/Abstract]) OR (Cost Benefit Data [Title/Abstract]) OR (Data, Cost-Benefit [Title/Abstract]) OR (Economic Evaluation [Title/Abstract]) OR (Economic Evaluations [Title/Abstract]) OR (Evaluation, Economic [Title/Abstract]))) OR ((Cost-Minimization Analysis [Title/Abstract]) OR (Analyses, Cost-Minimization [Title/Abstract]) OR (Analysis, Cost-Minimization [Title/Abstract]) OR (Cost Minimization Analysis [Title/Abstract]) OR (Cost-Minimization Analyses [Title/Abstract]))) |
| #3 | #1 AND #2 |

The Embase search yielded 52,498 records using the strategy.

| **Search ID** | **Search Details** |
| --- | --- |
| #1 | 'cost effectiveness analysis'/exp OR 'cost effectiveness analysis' OR 'cost benefit analysis'/exp OR 'cost benefit analysis' OR 'cost minimization analysis'/exp OR 'cost minimization analysis' OR 'cost utility analysis'/exp OR 'cost utility analysis' |
| #2 | 'drug administration'/exp OR 'drug administration' OR 'drug dosage form'/exp OR 'drug dosage form' |
| #3 | 'capsules' ab,ti OR 'capsule':ab,ti OR 'microcapsules':ab,ti OR 'microcapsule':ab,ti OR 'nasal sprays':ab,t OR 'sprays, nasal':ab,ti OR 'nasal mist': ab,ti OR 'mist, nasal':ab,ti OR 'nasal aerosol':ab,ti OR'aerosol, nasal' :ab,ti OR 'nasal spray':ab,ti OR 'spray, nasal':ab,ti OR 'oral sprays':ab,ti OR 'sprays, oral':ab,ti OR 'oral spray':ab,ti OR 'spray, oral':ab,ti OR 'emulsions':ab,ti OR 'emulsion':ab,ti OR 'fat emulsions, intravenous' :ab,ti OR 'emulsions, intravenous fat':ab,ti OR 'intravenous fat emulsions': ab,ti OR 'lipid emulsions, intravenous':ab,ti OR 'emulsions, intravenous lipid':ab,ti OR 'intravenous lipid emulsions':ab,ti OR 'intravenous lipid emulsion':ab,ti OR 'emulsion, intravenous lipid':ab,ti OR'lipid emulsion, intravenous':ab,ti OR 'intravenous fat emulsion':ab,ti OR 'emulsion, intravenous fat' :ab,ti OR 'fat emulsion, intravenous' :ab,ti OR 'liniments':ab,ti OR 'ointments' :ab,ti OR 'ointment':ab,ti OR 'unguents':ab,ti OR 'salve':ab,ti OR 'salves':ab,ti OR 'unguent':ab,ti OR'pastes':ab,ti OR 'paste':ab,ti OR 'skin ointment' :ab,ti OR 'ointment, skin':ab,ti OR 'powders':ab,ti OR 'powder':ab,ti OR 'suppositories':ab,ti OR 'vaginal suppositories':ab,ti OR 'suppositories, aginal':ab,ti OR 'vaginal suppository':ab,ti OR 'suppository, vaginal':ab,ti OR 'rectal suppositories':ab,ti OR 'suppositories, rectal':ab,ti OR 'tablets':ab,ti OR 'tablet':ab,ti OR 'vaginal creams, foams, and jellies':ab,ti OR 'vaginal creams, foams and jellies':ab,ti OR 'vaginal tablet':ab,ti OR 'tablet, vaginal':ab,ti OR 'tablets, vaginal':ab,ti OR 'vaginal tablets':ab,ti OR 'vaginal jelly':ab,ti OR 'jellies, vaginal':ab,ti OR 'jelly, vaginal':ab,ti OR 'vaginal jellies':ab,ti OR 'vaginal gel':ab,ti OR 'gel, vaginal':ab,ti OR 'gels, vaginal':ab,ti OR 'vaginal gels':ab,ti OR 'vaginal creams':ab,ti OR 'cream, vaginal':ab,ti OR 'creams, vaginal':ab,ti OR 'vaginal cream':ab,ti OR 'vaginal foams':ab,ti OR 'foam, vaginal':ab,ti OR 'foams, vag ginal':ab,ti OR 'vaginal foam':ab,ti |
| #4 | 'cost effectiveness analysis'/exp OR 'cost effectiveness analysis' OR 'cost benefit analysis'/exp OR 'cost benefit analysis' OR 'cost minimization analysis'/exp OR 'cost minimization analysis' OR 'cost utility analysis'/exp OR 'cost utility analysis' |
| #5 | 'cost-effectiveness analysis':ab,ti OR 'analysis, cost-effectiveness':ab,ti OR 'cost effectiveness analysis':ab,ti OR 'cost effectiveness':ab,ti OR 'effectiveness, cost':ab,ti OR 'cost effectiveness ratio':ab,ti OR 'cost effectiveness ratios':ab,ti OR 'effectiveness ratio, cost':ab,ti OR 'ratio, cost effectiveness';ab,ti |
| #6 | 'analysis, cost-effectiveness':ab,ti OR 'cost effectiveness analysis':ab,ti OR 'cost effectiveness:ab,ti' OR 'effectiveness, cost':ab,ti OR 'cost effectiveness ratio':ab,ti OR 'cost effectiveness ratios':ab,ti OR 'effectiveness ratio, cost' :ab,ti OR 'ratio, cost effectiveness':ab,ti OR 'cost-effectiveness analysis':ab,ti |
| #7 | 'cost-benefit analysis':ab,ti OR 'analysis, cost-benefit':ab.ti OR 'cost-benefit analyses':ab,ti OR 'cost benefit analysis':ab,ti OR 'analyses, cost benefit' :ab, ti OR 'analysis, cost benefit':ab,ti OR 'cost benefit analyses':ab,ti OR 'cost-utility analysis':ab,ti OR'analysis, cost-utility':ab,ti OR 'cost utility analysis':ab,ti OR 'cost-utility analyses':ab,ti OR 'cost benefit":ab,ti OR 'costs and benefits':ab,ti OR 'benefits and costs' :ab,ti OR 'cost and benefit':ab,ti OR 'benefit and cost':ab,ti OR 'marginal analysls':ab,ti OR 'analysis, marginal':ab,ti OR 'marginal analyses':ab,ti OR 'cost-benefit data':ab,ti OR 'cost benefit data': ab,ti OR 'data, cost-benefit': ab ti OR 'economic evaluation': ab,ti OR 'economic evaluations':ab,ti OR 'evaluation, economic':ab,ti |
| #8 | #1 OR #4 OR #5 OR #6 OR #7 |
| #9 | #2 OR #3 |
| #10 | #8 AND #9 |
| #11 | #10 AND 'article'/it |

# Table 2 Summaries of Main Characters of Included Studies

| **Study** | **Indication** | **ICD-11 Classification** | **Patient** | **Active Pharmaceutical Ingredient** | **Dosage Form (Intervention vs Control)** | **Route Comparison (Intervention vs Control)** | **Type of Analysis** | **Funding Type** |
| --- | --- | --- | --- | --- | --- | --- | --- | --- |
| Abramson, 2019, USA[1] | Type 2 diabetes | Endocrine, nutritional or metabolic diseases | The study subjects were 50-year-old American men and women with type 2 diabetes and an HbA1c value of 8.0%. | Semaglutide | Capsule vs Solution for injection | Oral vs Subcutaneous injection | Cost-Effectiveness Analysis | Non-Profit Organizations |
| Abushanab, 2021, Qatar[2] | Patent Ductus Arteriosus (PDA) in preterm infants | Diseases of the circulatory system | Preterm infants in Qatar (less than 37 weeks of gestation) with hemodynamically significant PDA | Ibuprofen | Oral suspension vs Solution for injection | Oral vs Intramuscular injection | Cost-Effectiveness Analysis | Non-Profit Organizations |
| Alsina, 2022, Spain[3] | Primary immunodeficiency diseases | Certain disorders involving the immune mechanism | PID patients in Spain requiring immune globulin replacement therapy | Immunoglobulin | Solution for infusion vs Solution for subcutaneous injection | Subcutaneous injection vs Intravenous injection | Cost Minimization Analysis | Profit Organizations |
| Altini, 2020, Italy[4] | Non-Hodgkin lymphoma | Neoplasms | Patients with Non-Hodgkin lymphoma | Rituximab | Solution for subcutaneous injection vs Solution for infusion | Subcutaneous injection vs Intravenous injection | Cost Minimization Analysis | Profit Organizations |
| Altini, 2020, Italy[4] | HER2-positive breast cancer | Neoplasms | Patients with HER2-positive breast cancer | Trastuzumab | Solution for subcutaneous injection vs Powder for concentrate for solution for infusion | Subcutaneous injection vs Intravenous injection | Cost Minimization Analysis | Profit Organizations |
| Alonso Torres, 2023, Spain[5] | Multiple Sclerosis | Diseases of the nervous system | Patients with highly active RRMS | Natalizumab | Solution for injection vs Concentrate for solution for infusion | Subcutaneous injection vs Intravenous injection | Cost Minimization Analysis | Profit Organizations |
| Ascher Svanm 2012, USA[6] | Schizophrenia | Mental, behavioural or neurodevelopmental disorders | Adult patients in the United States with schizophrenia | Olanzapine | Orally Disintegrating Tablet vs Standard Oral Tablet | Oral vs Oral | Cost-Effectiveness Analysis | Profit Organizations |
| Ascher Svanm 2012, USA[6] | Schizophrenia | Mental, behavioural or neurodevelopmental disorders | Adult patients with schizophrenia in the United States | Risperidone | Orally Disintegrating Tablet vs Standard Oral Tablet | Oral vs Oral | Cost-Effectiveness Analysis | Profit Organizations |
| Ascher Svanm 2012, USA[6] | Schizophrenia | Mental, behavioural or neurodevelopmental disorders | Adult patients with schizophrenia in the United States | Aripiprazole | Orally Disintegrating Tablet vs Standard Oral Tablet | Oral vs Oral | Cost-Effectiveness Analysis | Profit Organizations |
| A˚se Bjo¨rstad, 2017, Sweden[7] | House Dust Mite Allergy | Diseases of the respiratory system | Moderate to severe dust mite allergic rhinitis, with/without asthma | House Dust Mite (HDM) Allergen Extract | Sublingual tablet vs Subcutaneous injection | Sublingual administration vs Subcutaneous injection | Cost Minimization Analysis | Profit Organizations |
| Carter, J.A. 2017, USA[8] | Opioid Use Disorder | Mental, behavioural or neurodevelopmental disorders | Clinically stable adults with opioid use disorder in the United States | Buprenorphine | Subdermal implant vs Sublingual tablet | Subdermal implantation vs Sublingual administration | Cost-Effectiveness Analysis | Profit Organizations |
| C Mylonas, 2014, Greece[9] | HER2+ Breast cancer | Neoplasms | Greek women with HER2-positive early/metastatic breast cancer | Trastuzumab | Solution for subcutaneous injection vs Powder for concentrate for solution for infusion | Subcutaneous injection vs Intravenous injection | Cost Minimization Analysis | Profit Organizations |
| Davies, L. 1996, UK[10] | AIDS-related cytomegalovirus (CMV) retinitis | Certain infectious or parasitic diseases | AIDS-associated CMV retinitis in UK, Europe, Australia | Ganciclovir | Tablet vs Intravenous injection | Oral vs Intravenous injection | Cost Minimization Analysis | Profit Organizations |
| Ellis, A.K. 2019, Canada[11] | House dust mite (HDM) induced allergic rhinitis | Diseases of the respiratory system | Canadian patients aged 18-65 years with moderate to severe seasonal allergic rhinitis | HDM Allergen Extract | Sublingual tablet vs Solution for subcutaneous injection | Sublingual administration vs Subcutaneous injection | Cost Minimization Analysis | Profit Organizations |
| Ellis, A.K. 2021, Canada[12] | Seasonal allergic rhinitis (AR) induced by tree pollen | Diseases of the respiratory system | Canadian patients aged 18-65 years with moderate to severe seasonal allergic rhinitis | White Birch (Betula verrucosa) Pollen Allergen Extract | Sublingual tablet vs Subcutaneous injection | Sublingual administration vs Subcutaneous injection | Cost Minimization Analysis | Profit Organizations |
| Ellis, A.K. 2023, Canada[13] | Allergic rhinoconjunctivitis (ARC) due to ragweed pollen | Diseases of the respiratory system | Canadian children over 5 years old with ragweed pollen allergic rhino conjunctivitis | Short Ragweed (Ambrosia artemisiifolia) Allergen Extract | Sublingual tablet vs Subcutaneous injection | Sublingual administration vs Subcutaneous injection | Cost Minimization Analysis | Profit Organizations |
| Fargier, E. 2018, France[14] | Follicular Lymphoma | Neoplasms | Patients with follicular lymphoma receiving rituximab maintenance therapy | Rituximab | Solution for subcutaneous injection vs Solution for injection | Subcutaneous injection vs Intravenous injection | Cost-Consequence Analysis | None |
| Guo, S. 2009,USA[15] | Relapsing multiple sclerosis | Diseases of the nervous system | Adult patients with relapsing multiple sclerosis in the United States with an EDSS score of 0-5.5 | Interferon beta-1a | Solution for subcutaneous injection vs Solution for intramuscular injection | Subcutaneous injection vs Intramuscular injection | Cost-Effectiveness Analysis | Profit Organizations |
| Hu, S. 2021,China[16] | Type 1 Diabetes Melitus | Endocrine, nutritional or metabolic diseases | Chinese children aged 2-18 with type 1 diabetes | Insulin | Solution for subcutaneous infusion vs Solution for intravenous infusion | Subcutaneous injection vs Intravenous injection | Cost-Effectiveness Analysis | Non-Profit Organizations |
| Kalathr Raghu 2020,USA[17] | Anemia in children with chronic intestinal failure | Diseases of the blood or blood-forming organs | Children with chronic intestinal failure | Ferrous sulfate | Solution for intravenous injection vs Oral solution | Intravenous injection vs Oral | Cost-Effectiveness Analysis | Non-Profit Organizations |
| Lazzaro, C. 2014, Italy[18] | Chronic inflammatory demyelinating polyneuropathy | Diseases of the nervous system | Italian CIDP patients | Immunoglobulin | Subcutaneous injection vs Intravenous injection | Subcutaneous injection vs Intravenous injection | Cost Minimization Analysis | Profit Organizations |
| Lazzaro, C.2022, France[19] | Open-angle glaucoma (OAG) or ocular hypertension (OHT) with concomitant ocular surface disease (OSD) | Diseases of the eye and adnexa | The average age of patients with open-angle glaucoma (OAG) or elevated intraocular pressure (OHT) and ocular surface disease (OSD) in France is 47.31 years | Latanoprost | Cationic ophthalmic emulsion vs Eye drops, solution | Ocular use vs Ocular use | Cost Utility Analysis | Profit Organizations |
| Lazzaro, C. 2022, Germany[20] | Open-angle glaucoma or ocular hypertension with concomitant ocular surface disease | Diseases of the eye and adnexa | In Germany, the average age of patients with open-angle glaucoma or elevated intraocular pressure with ocular surface disease is 44.68 years. | Latanoprost | Cationic ophthalmic emulsion vs Eye drops, solution | Ocular use vs Ocular use | Cost Utility Analysis | Profit Organizations |
| Lazzaro, C. 2023, Italy[21] | Open angle glaucoma/ocular hypertension | Diseases of the eye and adnexa | Italian patients with Open-Angle Glaucoma, Ocular Hypertension, and Ocular Surface Disease, average age 57–60 years | Latanoprost | Cationic ophthalmic emulsion vs Eye drops, solution | Ocular use vs Ocular use | Cost Utility Analysis | Profit Organizations |
| Lee, V.W.Y. 2023, Hong Kong[22] | Breast cancer | Neoplasms | Hong Kong female patients with HER2-positive breast cancer | Trastuzumab | Solution for subcutaneous injection vs Solution for injection | Subcutaneous injection vs Intravenous injection | Cost Minimization Analysis | None |
| Lin, Z. 2020, China[23] | Schizophrenia | Mental, behavioural or neurodevelopmental disorders | Chinese schizophrenia patients | Aripiprazole | Orally Disintegrating Tablet vs Standard Oral Tablet | Oral vs Oral | Cost-Effectiveness Analysis | Profit Organizations |
| Liu, C. 2024, China[24] | Amyotrophic lateral sclerosis (ALS) | Diseases of the nervous system | Patients with ALS (starting age: 54 years) | Edaravone | Sublingual tablet vs Solution for intravenous injection | Sublingual administration vs Intravenous injection | Cost Utility Analysis | None |
| Marchetti, A. 2009, USA[25] | Acute acetaminophen poisoning | Injury, poisoning or certain other consequences of external causes | American patients with acute acetaminophen poisoning | Acetaminophen | Solution for intravenous infusion vs Oral solution | Intravenous injection vs Oral | Cost Minimization Analysis | Profit Organizations |
| Martin, A. 2013, USA[26] | Primary immune deficiency | Certain disorders involving the immune mechanism | Canadian patients with primary immunodeficiency | Immunoglobulin | Solution for subcutaneous injection vs Solution for intravenous infusion | Subcutaneous injection vs Intravenous injection | Cost Minimization Analysis | Profit Organizations |
| O’Brien, G.L. 2019, Ireland[27] | HER2-positive breast cancer | Neoplasms | Patients with HER2-positive breast cancer | Trastuzumab | Subcutaneous injection vs Intravenous injection | Subcutaneous injection vs Intravenous injection | Cost Minimization Analysis | Profit Organizations |
| O’Cathail 2013, UK[28] | Gynecological malignancies | Injury, poisoning or certain other consequences of external causes | Patients in the UK and Canada with gynecological malignancies, primarily ovarian, endometrial, and cervical cancer. | Dexamethasone | Solution for intravenous injection vs Tablet | Intravenous injection vs Oral | Cost-Effectiveness Analysis | Non-Profit Organizations |
| Perraudin, C. 2020, Switzerland[29] | Chronic inflammatory demyelinating polyneuropathy | Diseases of the nervous system | Swiss chronic inflammatory demyelinating polyneuropathy in stable adult patients | Immunoglobulin | Subcutaneous injection vs Intravenous injection | Subcutaneous injection vs Intravenous injection | Cost Minimization Analysis | None |
| Rojas, L. 2020, Chile[30] | HER2-positive early breast cancer | Neoplasms | Female patients in Chile with HER2-positive early breast cancer | Trastuzumab | Solution for subcutaneous injection vs Solution for intravenous injection | Subcutaneous injection vs Intravenous injection | Cost Minimization Analysis | Profit Organizations |
| Rønborg, S. 2016, Denmark[31] | House dust mite respiratory allergic disease | Diseases of the respiratory system | Danish patients with moderate to severe dust mite allergic rhinitis and/or asthma | House Dust Mite (HDM) Allergen Extract | Sublingual tablet vs Solution for subcutaneous injection | Sublingual administration vs Subcutaneous injection | Cost Minimization Analysis | Profit Organizations |
| Rudis, M.I. 2004, USA[32] | Seizures with subtherapeutic phenytoin concentrations | Diseases of the nervous system | Patients with seizures and inadequate phenytoin concentrations in U.S. emergency departments | Phenytoin | Tablet vs Solution for intravenous injection | Oral vs Intramuscular injection | Cost-Effectiveness Analysis | Non-Profit Organizations |
| Saha, S. 2024, India[33] | Iron deficiency anemia among pregnant women | Diseases of the blood or blood-forming organs | Pregnant women in India with moderate to severe anemia between 14 and 18 weeks of gestation | Ferrous sulfate | Solution for intravenous injection vs Tablet | Intravenous injection vs Oral | Cost-Effectiveness Analysis | Non-Profit Organizations |
| Simoens, S. 2021, Belgium[34] | HER2-positive breast cancer | Neoplasms | Adjuvant therapy for women with HER2-positive breast cancer | Trastuzumab | Subcutaneous injection vs Intravenous injection | Subcutaneous injection vs Intravenous injection | Cost Minimization Analysis | None |
| Somerville 2003, USA[35] | Cytomegalovirus | Certain infectious or parasitic diseases | CMV disease in US, including AIDS and liver transplant patients | Ganciclovir | Tablet vs Solution for intravenous infusion | Oral vs Intravenous injection | Cost-Effectiveness Analysis | Profit Organizations |
| Sullivan, S.D. 1996, USA[36] | Cytomegalovirus Retinitis | Diseases of the eye and adnexa | Newly diagnosed cytomegalovirus retinitis in patients with AIDS | Ganciclovir | Tablet vs Intravenous formulation | Oral vs Intravenous injection | Cost Minimization Analysis | None |
| Vidal-Alaball 2006, UK[37] | Vitamin B12 deficiency | Endocrine, nutritional or metabolic diseases | Vitamin B12 deficiency patients in the UK | Vitamin B12 | Tablet vs Intramuscular injection | Oral vs Intramuscular injection | Cost Minimization Analysis | None |
| Wang, G.H.M. 2023, USA[38] | Schizophrenia | Mental, behavioural or neurodevelopmental disorders | 40-year-old American patient with schizophrenia | Paliperidone | Prolonged-release suspension for intramuscular injection vs Prolonged-release (extended-release) tablet | Intramuscular injection vs Oral | Cost-Effectiveness Analysis | None |

# Table 3 Methodological Features of Included Cost-Effectiveness and Cost-Utility Analyses

| **Study** | **Model Type** | **Perspective** | **Source of Safety and Efficacy Data** | **Types of Costs Included** | **Outcome Measure** | **Source of Cost Data** | **Source of Utility Data** |
| --- | --- | --- | --- | --- | --- | --- | --- |
| Abramson, 2019[1] | First-order Monte Carlo microsimulation model | Payer perspective | Equivalence clinical trial | Drug costs | Cost; Utility; Incremental Cost-Effectiveness Ratio | Market retail price and production cost | Literature |
| Abushanab, 2021[2] | Decision-Analytic Model | Hospital | Retrospective cohort study | Drug costs; Costs of managing adverse events; Diagnostic and laboratory costs; NICU Bed Costs | Cost; Success rate of Patent Ductus Arteriosus closure | The hospital’s Cerner medical database (2014–2018); The hospital’s Finance and Accounting Department | NA |
| Ascher Svanm 2012[6] | Monte Carlo Micro-simulation Model | Third-party payer perspective | Randomized Controlled Trial | Medication cost; Cost of stable patients; Inpatient and outpatient relapse costs; Cost of adverse events | Cost; Utility; Incremental Cost-Effectiveness Ratio | 2010 Net Wholesale Price； American healthcare economics research and literature | Literature |
| Carter, J.A. 2017[8] | Markov Model | American society perspective | Randomized Controlled Trial | Direct Medical Costs（Drug acquisition/administration costs；Treatment diversion/abuse costs；Newly Diagnosed HCV-Related Costs；Emergency room and hospitalization costs; Rehabilitation services costs; Pediatric poisoning costs);Indirect Non-Medical Costs (Criminal justice costs; Lost wages/work productivity; Out-of-pocket expenses) | Cost; Utility | Literature, observational studies, and administrative claims data | Literature |
| Guo, S. 2009[15] | Discrete Event Simulation | Healthcare payer perspective | Randomized Controlled Trial | Direct medical costs (Drug costs. Direct medical costs during relapses; Management costs during remission; Long-term management costs for secondary progressive multiple sclerosis; MRI examination costs) | Cost; Incremental Cost-Effectiveness Ratio | 2006 Red Book, literature and databases | NA |
| Hu, S. 2021[16] | IQVIA CORE Diabetes Model | A public health system perspective | Retrospective cohort study | Direct Medical Costs (Drug; insulin pump device; consumables; insulin; complication management) | Cost; Utility; Incremental Cost-Effectiveness Ratio | Local product prices; Literature review | Literature |
| Kalathr Raghu 2020[17] | Microsimulation Model | Healthcare perspective | Retrospective cohort data | Drug cost; Transfusion cost | Cost; Effectiveness | Federal pricing schedule and literature | NA |
| Lazzaro, C.2022[19] | Markov model | French medical system perspective | Randomized Controlled Trial | Direct Medical Costs (Drug; Treatment & Follow-up; Ocular Disease Management) | Cost; Utility; Incremental Cost-Utility Ratio | Ontario Nurses' Association Collective Agreement | Literature and Expert Opinion |
| Lazzaro, C. 2022[20] | Markov model | German medical system perspective | Randomized Controlled Trial | Direct Medical Costs (Drug; Treatment & Follow-up; Ocular Disease Management) | Cost; Utility; Incremental Cost-Utility Ratio | Manufacturer's Price Data for Pharmaceuticals; German Physician Fee Schedule; Diagnosis-Related Group Tariffs | Literature and Expert Opinion |
| Lazzaro, C. 2023[21] | Markov model | Italian National Health Service (INHS) | Randomized Controlled Trial | Direct Medical Costs (Drug; Treatment & Follow-up; Ocular Disease Management) | Cost; Utility; Incremental Cost-Utility Ratio | Ontario Health Insurance Plan Payment Schedule | Literature and Expert Opinion |
| Lin, Z. 2020[23] | Discrete Event Simulation | Chinese healthcare payers | Randomized Controlled Trial and Expert Opinion | Direct Medical Costs (Drug costs; Hospitalization costs; Outpatient costs; Laboratory and procedure costs; Costs of managing adverse events) | Cost; Utility | Average drug bid prices from 2017 to 2019 and literature | Literature |
| Liu, C. 2024[24] | Markov model | Societal perspective | Assumption | Direct medical costs (Drug costs; Catheter insertion and maintenance costs; Adverse event management costs); Direct non-medical costs (Transportation costs); Indirect costs (costs of work delay/lost productivity) | Cost; Utility; Incremental Cost-Effectiveness Ratio | The publicly available government service price list of China in 2023 and the per capita annual disposable income in 2022 | Literature |
| O’Cathail 2013[28] | Markov Monte-Carlo model | Social perspective | Centrally collected prospective data | Drug costs; Medical service costs; Costs related to managing allergic reactions | Cost; Life year; Incremental Cost-Utility Ratio | 2010-2011 NHS Reference Costs and British National Formulary 2011 Edition | NA |
| Rudis, M.I. 2004[32] | Decision tree model | Healthcare system | Randomized Controlled Trial | Drug costs; Material costs (Normal saline; Needles; Syringes); Labor costs (Time costs of nurses and doctors); Costs of managing adverse events | Cost; Average time to safe discharge; Number of adverse events | Prospective, randomized controlled clinical trial and Hospital administrative database | NA |
| Saha, S. 2024[33] | Decision tree model | social perspective | Prospective cohort follow-up | Treatment costs; Consumables costs; Medical resource utilization; Patient out-of-pocket expenses; Productivity loss | Cost; Utility; Incremental Cost-Utility Ratio; | Government-approved rate contracts; Local procurement prices; Facility consumption lists; Administrative records; On-site interviews | Baseline and follow-up outcomes in the study |
| Somerville 2003[35] | Markov model | Social Perspective and Patient Perspective | Randomized Controlled Trial | Direct Medical Costs (Drug costs; Diagnostic costs; Acute graft rejection management costs) | Cost; Utility; Incremental Cost-Effectiveness Ratio | U.S. government data from 1994 and 1995 | Literature |
| Wang, G.H.M. 2023[38] | Markov model | Third-party payer perspective | Randomized Controlled Trial | Direct Medical Costs (Drug costs; Relapse-related medical costs; Injection; follow-up) | Cost; Utility; Incremental Cost-Effectiveness Ratio | Veterans Affairs Federal Supply Schedule and Centers for Medicare & Medicaid Services | Literature |

# Table 4 Key Characteristics and Main Findings of Included Cost-Effectiveness and Cost-Utility Analyses

| **Study** | **Time horizon** | **Cycle length** | **Uncertainty Analysis** | **Discount** | **Willingness-to-Pay Threshold** | **Results** |
| --- | --- | --- | --- | --- | --- | --- |
| Abramson, 2019[1] | Lifetime | 1 year | Two-way sensitivity analysis and probabilistic sensitivity analysis | 3%/year | $85,000-$110,000/QALY | Male：ICER=$85000/QALY  Female: ICER=$92000/QALY  (2019) |
| Abushanab, 2021[2] | NA | NA | Single-factor sensitivity analysis and probabilistic sensitivity analysis | NA | NA | Oral ibuprofen PDA Closure Rate=64%  Intravenous ibuprofen PDA Closure Rate=36%  Δ Cost=$13356  (2021) |
| Ascher Svanm 2012[6] | 1 year | 3 months | Single-factor sensitivity analysis and probabilistic sensitivity analysis | NA | $50,000/QALY | ICER=$19643/QALY  (2010) |
| Ascher Svanm 2012[6] | 1 year | 3 months | Single-factor sensitivity analysis and probabilistic sensitivity analysis | NA | $50,000/QALY | ICER=$157000/QALY  (2010) |
| Ascher Svanm 2012[6] | 1 year | 3 months | Single-factor sensitivity analysis and probabilistic sensitivity analysis | NA | $50,000/QALY | ICER=$21077/QALY  (2010) |
| Carter, J.A. 2017[8] | 1 year | 1 month | Single-factor sensitivity analysis and probabilistic sensitivity analysis | NA | $50,000/QALY | Buprenorphine subcutaneous implant dominant  (2016) |
| Guo, S. 2009[15] | 4 years | 1 year | Single-factor sensitivity analysis and probabilistic sensitivity analysis | 3%/year | NA | Subcutaneous injections cost $10,755 more than intramuscular injections for each relapse avoided.  (2016) |
| Hu, S. 2021[16] | 60 years | 1 year | Single-factor sensitivity analysis ； Probabilistic sensitivity analysis；Scenario analysis | 5%/year | ¥186,423-¥372,846/QALY | ICER=¥161815 /QALY  (2021) |
| Kalathr Raghu 2020[17] | 1 year | NA | Single-factor sensitivity analysis and probabilistic sensitivity analysis | NA | $871 per occurrence | Parenteral iron costs $6,600 more than oral iron for each transfusion avoided.  (2018) |
| Lazzaro, C.2022[19] | 5 years | 1 year | Single-factor sensitivity analysis and probabilistic sensitivity analysis | 2.5%/year | €30,000- €50,000/QALY | ICUR=€21.26/QALY  (2020) |
| Lazzaro, C. 2022[20] | 5 years | 1 year | Single-factor sensitivity analysis and probabilistic sensitivity analysis | 3%/year | €30,000/QALY | ICUR=€573.81/QALY  (2020) |
| Lazzaro, C. 2023[21] | 5 years | 1 year | Single-factor sensitivity analysis and probabilistic sensitivity analysis | 3%/year | €25,000-€40,000/QALY | ICUR=€647.65/QALY  (2020) |
| Lin, Z. 2020[23] | 1 year | NA | Single-factor sensitivity analysis and probabilistic sensitivity analysis | NA | $28112/QALY | ODT: QALY=0.7282；Cost=$1423  SOT: QALY=0.7112；Cost=$2215  (2019) |
| Liu, C. 2024[24] | 20 years | 3 months | Single-factor sensitivity analysis and probabilistic sensitivity analysis | 5%/year | ¥268074/QALY | ICER=￥372,648.24/QALY  (2023) |
| O’Cathail 2013[28] | Lifetime | 1 week | NA | 3%/year | £30,000/QALY | ICER =£20647/year  (2011) |
| Rudis, M.I. 2004[32] | NA | NA | Single-factor sensitivity analysis and probabilistic sensitivity analysis | NA | Low willingness-to-pay threshold (< $2/hour)； Moderate willingness-to-pay threshold ($2-$14/hour)；High willingness-to-pay threshold (> $14/hour) | Intravenous phenytoin costs $3.90 more than oral phenytoin for each additional hour of hospitalization time saved.  (2003) |
| Saha, S. 2024[33] | 1 year | NA | Single-factor sensitivity analysis | NA | $20081/QALY | ICUR=$9.84/QALY  (2023) |
| Somerville 2003[35] | 1 year | NA | Probabilistic sensitivity analysis | NA | NA | Δ Cost=$6781  (1995) |
| Wang, G.H.M. 2023[38] | 5 years | 3 months | Single-factor sensitivity analysis and probabilistic sensitivity analysis | 3%/year | $50,000/QALY | ICER=$932698/QALY  (2022) |
| Wang, G.H.M. 2023[38] | 5 years | 3 months | Single-factor sensitivity analysis and probabilistic sensitivity analysis | 3%/year | $50,000/QALY | ICER=$185402/QALY  (2022) |
| Wang, G.H.M. 2023[38] | 5 years | 3 months | Single-factor sensitivity analysis and probabilistic sensitivity analysis | 3%/year | $50,000/QALY | ICER=$179089/QALY  (2022) |

# Table 5 Key Characteristics of Included Cost-Minimization Analyses Studies

| **Study** | **Perspective** | **Source of Safety and Efficacy Data** | **Types of Costs Included** | **Outcome Measure** | **Source of Cost Data** |
| --- | --- | --- | --- | --- | --- |
| Alsina, 2022[3] | Spanish National Healthcare System and Social Perspectives | Randomized controlled clinical trials | Direct costs (medication, hospital administration, SCIG training, medication distribution, pre-treatment medication); indirect costs (work absence, lost productivity) | Total cost; Drug cost; non-pharmaceutical direct medical cost; Indirect costs; Administration time | Spanish Drug Price Database |
| Altini, 2020[4] | Healthcare systems and social perspectives | Randomized controlled clinical trials | Direct: medication, staff, consumables; indirect: patient/caregiver lost work time. | Total cost; Drug cost; non-pharmaceutical direct medical cost; Indirect costs | Hospital database, interviews |
| Alonso Torres, 2023[5] | Spanish National Health System and societal perspective | Equivalence Clinical trial | Direct costs (medication preparation, administration, staff time, facility use); indirect costs (productivity loss for patients and caregivers). | Total cost; non-pharmaceutical direct medical cost; Indirect cost (patient + caregiver); Administration time | Official regional bulletin of Spain (2021) |
| A˚se Bjo¨rstad, 2017[7] | Societal perspective and Healthcare perspective | Randomized controlled clinical trials | Direct medical costs (drug, healthcare personnel, facility); direct non-medical costs (patient travel); indirect costs (productivity loss). | Total cost; Drug cost; non-pharmaceutical direct medical cost; Indirect cost (patient + caregiver) | Swedish public price list |
| C Mylonas, 2014[9] | Hospital perspective | Randomized controlled clinical trials | Drug acquisition, administration, hospital overhead costs. | Total cost; Drug cost; non-pharmaceutical direct medical cost; Indirect cost | Greek healthcare system |
| Davies, L. 1996[10] | National Health Service Perspective | Randomized controlled clinical trials | Direct medical costs (hospitalization, outpatient, drugs, consumables, laboratory tests). | Administration cost; Hospital management cost; Total cost | National Health Service |
| Ellis, A.K. 2019[11] | Societal Perspective | Randomized controlled clinical trials | Direct costs: drug costs, medical service costs; Indirect costs: patient productivity loss and transportation costs due to treatment | Total cost; Drug cost; non-pharmaceutical direct medical cost; Indirect costs | Ontario Public Pharmacy Program, Ontario Ministry of Health and Long-Term Care |
| Ellis, A.K. 2021[12] | Societal Perspective | Equivalence assumption | Direct: medication, medical services; indirect: travel expenses, lost work time. | Total cost; Drug cost; non-pharmaceutical direct medical cost; Indirect costs | Ontario public drug plan and Quebec health insurance, Ontario and Quebec medical fee schedules. |
| Ellis, A.K. 2023[13] | Public Payer Perspective | Randomized controlled clinical trials | Medication costs: Healthcare provider costs (doctor fees, nurse fees, delivery costs); Other indirect costs (patient/caregiver time) | Total cost; Drug cost; non-pharmaceutical direct medical cost | Régie de l'assurance maladie du Québec |
| Lazzaro, C. 2014[18] | Societal perspective | Previous research | Medical costs (medication, management drugs, staff time, consumables); non-medical (transportation, parking, lost work/leisure time). | Total cost; Drug cost; non-pharmaceutical direct medical cost; Indirect costs | Expert opinions, Italian published literature |
| Lee, V.W.Y. 2023[22] | Hospital Perspective | Randomized controlled clinical trials | Drug procurement, medical resources (nursing and pharmacist time), outpatient, and chemotherapy-related costs. | Total cost; Drug cost; non-pharmaceutical direct medical cost; Administration time | Pharmaceutical Manufacturers and Hong Kong Government Gazette 2017. |
| Marchetti, A. 2009[25] | Provider perspective | Retrospective cohort study | Direct medical costs (ER visits, ICU/ward hospitalizations, medications, treatment of hepatotoxicity, acute liver failure, liver transplantation). | Total cost; Drug cost; non-pharmaceutical direct medical cost | University of Medicine and Dentistry of New Jersey |
| Martin, A. 2013[26] | Healthcare System Perspective | Randomized controlled clinical trials and literature review | Direct medical costs (consumables costs), Human resource costs (training and follow-up costs for nurses/managers) | Total cost | British Columbia Ministry of Health, Canada |
| O’Brien, G.L. 2019[27] | Healthcare provider perspective | Randomized controlled clinical trials | Direct medical costs: drug costs, medication costs, patient visit costs | Total cost; Drug cost; non-pharmaceutical direct medical cost; Indirect costs | Irish medical institutions |
| Perraudin, C. 2020[29] | Societal perspective | Randomized controlled clinical trials | Direct medical (hospital services, home treatment materials, training, follow-up); direct non-medical (patient transportation); indirect (lost productivity). | Total cost; Drug cost; non-pharmaceutical direct medical cost; Indirect costs | Public prices for medicines in the Swiss market |
| Rojas, L. 2020[30] | Medical institution perspective and social perspective | Randomized controlled clinical trials | Direct medical costs (medication, equipment/consumables, pharmacist and nurse time, infusion chair time); indirect medical costs (treatment, testing, hospitalization for serious adverse reactions). | Total cost; Drug cost; non-pharmaceutical direct medical cost; Indirect costs | Nuestra Señora de la Esperanza Cancer Center |
| Rønborg, S. 2016[31] | Healthcare system | Indirect comparison | Direct costs (Medication costs, Doctor visit costs) Indirect costs (Patient productivity loss, transportation costs) | Total cost; Drug cost; non-pharmaceutical direct medical cost; Indirect costs | Danish market prices and medical association fees |
| Simoens, S. 2021[34] | Hospital perspective | Randomized controlled clinical trials | Direct costs (Drug costs, Healthcare staff time costs, Consumables costs) | Total cost; Drug cost; non-pharmaceutical direct medical cost | Belgian price list |
| Sullivan, S.D. 1996[36] | Medicaid payer perspective | Cohort observational study | Direct medical costs: drug costs, nursing costs, monitoring costs, and costs of treating adverse events. | Total cost; Drug cost; non-pharmaceutical | 1993 U.S. Medical Services Medicaid Reimbursement Database |
| Vidal-Alaball 2006[37] | National Health Service Perspective | Observational studies and systematic reviews | Direct costs (medication, injection materials, lab monitoring, nursing time, home visits). | Total cost; Drug cost; non-pharmaceutical direct medical cost | Literature review, British National Formulary |

**Table 6 Annual Costs and Cost Differences in Included Cost-Minimization Analyses Studies**

| **Study** | **Intervention Group Annual Cost (2024 US$)** | **Control Group Annual Cost (2024 US$)** | **Total cost Absolute Difference** | **Total cost Relative Difference** |
| --- | --- | --- | --- | --- |
| Alsina, 2022[3] | Direct Nondrug cost: $ 27041.79  Indirect cost: $ 140.42 | Direct Nondrug cost: $ 34312.83  Indirect cost: $ 885.9 | -$8016.52 | -22.78% |
| Altini, 2020[4]  (Trastuzumab) | Drug cost: $ 45874.9  Direct Nondrug cost: $ 2189.03  Indirect cost: $ 219.4 | Drug cost: $ 48035.68  Direct Nondrug cost: $ 2288.76  Indirect cost: $ 317.47 | -$2358.58 | -4.66% |
| Altini, 2020[4]  (Rituximab) | Drug cost: $19446.97  Direct Nondrug cost: $1524.18  Indirect cost: $49.86 | Drug cost: $20776.68  Direct Nondrug cost: $2084.32  Indirect cost: $59.84 | -**$**1899.83 | -8.29% |
| Alonso Torres, 2023[5] | Direct Nondrug cost: $ 1093.33  Indirect cost: $1065.36 | Direct Nondrug cost: $733.02  Indirect cost: $2200.45 | -**$**3574.39 | -62.35% |
| A˚se Bjo¨rstad, 2017[7] | Drug cost: $197.05  Direct Nondrug cost: $61.75 | Drug cost: $47.37  Direct Nondrug cost: $554.67 | **-$**343.24 | -57.01% |
| C Mylonas, 2014[9] | Drug cost: $43927.61  Direct Nondrug cost: $135.78  Indirect cost: $1125.05 | Drug cost: $45085.51  Direct Nondrug cost: $505.07  Indirect cost: $130.16 | -$532.30 | -1.16% |
| Ellis, A.K. 2019[11] | Drug cost: $3651.27  Direct Nondrug cost: $361.88  Indirect cost: $101.33 | Drug cost: $1094.28  Direct Nondrug cost: $4050.7  Indirect cost: $1860.05 | -**$**2890.55 | -41.26% |
| Ellis, A.K. 2021[12] | Drug cost: **$**829.38  Direct Nondrug cost: **$**99.57  Indirect cost: **$**107.53 | Drug cost: $259.87  Direct Nondrug cost: $481.9  Indirect cost: $664.1 | -**$**369.39 | -26.27% |
| Ellis, A.K. 2023[13] | Drug cost: **$**2064.18  Direct Nondrug cost: **$**255.38 | Drug cost: $748.95  Direct Nondrug cost: $2343.67 | -**$**773.06 | -25.00% |
| Fargier, E. 2018[14] | Drug cost: $17669.74  Direct Nondrug cost: $107.01 | Drug cost: $18523.1  Direct Nondrug cost: $337.98 | -**$**1084.33 | -5.75% |
| Lazzaro, C. 2014[18] | Drug cost: $ 79775.3  Direct Nondrug cost: $ 2669.57  Indirect cost: $ 2365.75 | Drug cost: $ 74993.44  Direct Nondrug cost: $ 5640.64  Indirect cost: $ 6506.75 | -$2330.21 | -2.67% |
| Lee, V.W.Y. 2023[22] | Drug cost: $ 41950.13  Direct Nondrug cost: $ 4841.71 | Drug cost: $ 54006.9  Direct Nondrug cost: $ 5488.7 | -$12703.76 | -21.35% |
| Martin, A. 2013[26] | Drug cost: $ 278.08  Direct Nondrug cost: $733.02 | Drug cost: $ 225.72  Direct Nondrug cost: $2858.71 | -$2073.33 | -67.22% |
| O’Brien, G.L. 2019[27] | Drug cost: $ 50068.57  Direct Nondrug cost: $ 884.27  Indirect cost: $ 97.73 | Drug cost: $ 50791.90  Direct Nondrug cost: $ 2504.13  Indirect cost: $ 354.73 | -$2600.19 | -4.85% |
| Perraudin, C. 2020[29] | Drug cost: $ 69058.88  Direct Nondrug cost: $ 9409.62  Indirect cost: $ 2202.91 | Drug cost: $ 83627.27  Direct Nondrug cost: $ 13574.24  Indirect cost: $ 8035.78 | -$24565.88 | -23.34% |
| Rojas, L. 2020[30] | Drug cost: $ 90866.00  Direct Nondrug cost: $ 5462.00 | Drug cost: $ 96773.67  Direct Nondrug cost: $ 7303 | -$7748.67 | -7.45% |
| Rønborg, S. 2016[31] | Drug cost: $460.87  Direct Nondrug cost: $46.84  Indirect cost: $606.99 | Drug cost: $197.89  Direct Nondrug cost: $428  Indirect cost: $1106.16 | -**$**617.35 | -35.64% |
| Simoens, S. 2021[34] | Drug cost: $23165.28  Direct Nondrug cost: $0 | Drug cost: $23165.28  Direct Nondrug cost: $1380.76 | -$1380.76 | -5.63% |
| Vidal-Alaball 2006[37] | Drug cost: $57.26  Direct Nondrug cost: $210 | Drug cost: **$**22.05  Direct Nondrug cost: **$**103.43 | **$**141.78 | 112.99% |

Note: All cost data were converted to 2024 US dollars by first adjusting for inflation to the target year in the original currency, and then converting to US dollars using purchasing power parity (PPP) exchange rates from the IMF dataset. Additionally, the “Total cost Absolute Difference” is calculated as the total cost of the intervention group minus the total cost of the control group.

The “Total cost Relative Difference” is calculated as: (Intervention group cost–Control group cost)/(Control group cost)×100%.

# Table 7 Formulation and Route of Administration Influence Economic Evaluation Results

| **Study** | **Source of Adherence Data** | **Sources of cost differences** | **Sources of effect differences** |
| --- | --- | --- | --- |
| Abramson, 2019[1] | Real-world study | The acquisition costs of oral active pharmaceutical ingredients differ from those of injectable active pharmaceutical ingredients. | The disutility associated with taking a daily oral tablet differs from that of receiving a weekly injection.  Differences in the frequency of injectable versus oral dosing lead to differences in treatment adherence. |
| Abushanab, 2021[2] | Not considered | Patients in the oral ibuprofen group required less mechanical ventilation and had shorter hospital stays, thereby reducing costs. | No difference. |
| Ascher Svanm 2012[6] | Observational study | Although orally disintegrating tablets have higher acquisition costs than standard oral tablets, they are associated with fewer hospitalisations and outpatient visits, resulting in lower total costs. | Orally disintegrating tablets are associated with lower outpatient relapse rates, lower inpatient relapse rates, and a higher proportion of patients without relapse. |
| Carter, J.A. 2017[8] | Randomized Controlled Trial | Subcutaneous administration is associated with higher drug acquisition costs but lower hospitalisation costs, resulting in lower direct medical costs. Criminal justice costs are lower, reducing non-medical costs, and wage and productivity losses are lower, reducing indirect costs. | No difference. |
| Guo, S. 2009[15] | Discontinuation rate in randomized controlled clinical trials | Subcutaneous injection is associated with lower relapse rates and fewer new disease events, resulting in lower direct medical costs. | Subcutaneous injection is associated with lower relapse rates, fewer non‑responders and fewer new disease events, resulting in better outcomes. |
| Hu, S. 2021[16] | Not considered | Subcutaneous insulin infusion requires insulin pumps and therefore has higher total direct medical costs than intravenous insulin. However, it is associated with lower rates of adverse events and lower costs for managing these events. | Subcutaneous insulin infusion has a lower incidence of adverse events than intravenous administration, reducing the disutility associated with adverse events. |
| Kalathr Raghu 2020[17] | Assumption | Parenteral iron supplementation requires fewer transfusions per patient per year than enteral iron supplementation, resulting in lower transfusion treatment costs. | No difference. |
| Lazzaro, C.2022[19] | Expert Opinion | Compared with conventional eye drops, cationic emulsions reduce follow‑up visit frequency, leading to lower direct medical costs. | No difference. |
| Lazzaro, C. 2022[20] | Expert Opinion | Compared with conventional eye drops, cationic emulsions reduce follow‑up visit frequency, leading to lower direct medical costs. | No difference. |
| Lazzaro, C. 2023[21] | Expert Opinion | Compared with conventional eye drops, cationic emulsions reduce follow‑up visit frequency, leading to lower direct medical costs. | No difference. |
| Lin, Z. 2020[23] | Clinician survey | Better adherence with orally disintegrating tablets leads to lower risks of relapse and hospitalisation. | Better adherence with orally disintegrating tablets leads to lower risks of relapse and hospitalisation. |
| Liu, C. 2024[24] | Not considered | Patients receiving sublingual administration have fewer hospital visits than those receiving intravenous administration, resulting in lower medical costs and lower direct non‑medical costs (such as travel costs). | Patients receiving sublingual edaravone have higher utility than those receiving intravenous edaravone. |
| O’Cathail 2013[28] | Assume full patient adherence | The incidence of severe allergic reactions is lower with oral dexamethasone than with injectable dexamethasone, resulting in lower costs. | The incidence of severe allergic reactions is lower with oral dexamethasone than with injectable dexamethasone. |
| Rudis, M.I. 2004[32] | Not considered | Oral phenytoin, compared with intravenous phenytoin, significantly reduces emergency department length of stay. | No difference. |
| Saha, S. 2024[33] | Prospective cohort follow-up | No difference. | Intravenous iron supplementation has higher adherence and fewer adverse events than oral iron supplementation. |
| Somerville 2003[35] | Not considered | Oral ganciclovir has a lower infection rate than intravenous ganciclovir, improves quality of life and reduces hospital length of stay, thereby using fewer healthcare resources and lowering costs. | Oral ganciclovir has a lower risk of infection and a lower incidence of adverse events than intravenous ganciclovir. |
| Wang, G.H.M. 2023[38] | Real-world data | Long‑acting injectable iron has higher drug acquisition costs than oral iron. | Long‑acting injectable iron performs better than oral iron in terms of relapse rates, hospitalisation rates, mortality and quality of life. |
| Alsina, 2022[3] | Not considered | Compared with intravenous administration, subcutaneous injection requires less travel time to hospital and less nursing time, leading to lower travel and time‑loss costs, lower caregiving and hospital management costs, and a lower required dose, which reduces drug acquisition costs. | No difference. |
| Altini, 2020[4] | Not considered | Compared with intravenous administration, subcutaneous injection requires less travel time to hospital and less nursing time, leading to lower travel and time‑loss costs, lower caregiving and hospital management costs, and a lower required dose, which reduces drug acquisition costs. | No difference. |
| Alonso Torres, 2023[5] | Not considered | For patients, subcutaneous natalizumab is more convenient than intravenous administration and reduces treatment time. | No difference. |
| A˚se Bjo¨rstad, 2017[7] | Not considered | Subcutaneous Dermatophagoides pteronyssinus immunotherapy requires a lower drug dose than intravenous administration, resulting in lower costs. | No difference. |
| C Mylonas, 2014[9] | Not considered | Compared with intravenous use, subcutaneous trastuzumab shifts costs away from administration‑ and hospital‑related resources while only slightly increasing drug acquisition costs. Overall, this results in lower total treatment expenditures and notable time savings for both patients and healthcare staff. | No difference. |
| Davies, L. 1996[10] | Not considered | Oral ganciclovir has lower drug acquisition and nursing costs than intravenous ganciclovir, resulting in lower total costs. | No difference. |
| Ellis, A.K. 2019[11] | Not considered | Sublingual Dermatophagoides pteronyssinus tablets have higher drug acquisition costs than subcutaneous injections, but they eliminate the need for frequent hospital visits, saving travel and time (productivity) costs and resulting in lower total costs. | No difference. |
| Ellis, A.K. 2021[12] | Not considered | Sublingual Dermatophagoides pteronyssinus tablets have higher drug acquisition costs than subcutaneous injections, but they eliminate the need for frequent hospital visits, saving travel and time (productivity) costs and resulting in lower total costs. | No difference. |
| Ellis, A.K. 2023[13] | Not considered | Sublingual Dermatophagoides pteronyssinus tablets have higher drug acquisition costs than subcutaneous injections, but they eliminate the need for frequent hospital visits, saving travel and time (productivity) costs and resulting in lower total costs. | No difference. |
| Lazzaro, C. 2014[18] | Not considered | Subcutaneous immunoglobulin, compared with intravenous immunoglobulin, reduces the need for informal care and decreases time lost due to treatment administration. | No difference. |
| Lee, V.W.Y. 2023[22] | Not considered | Subcutaneous trastuzumab requires less drug preparation and nursing time than intravenous administration, leading to lower drug acquisition and healthcare professional time costs. | No difference. |
| Marchetti, A. 2009[25] | Not considered | Intravenous N‑acetylcysteine is associated with shorter hospital stays and lower total inpatient nursing costs than oral N‑acetylcysteine. | No difference. |
| Martin, A. 2013[26] | Not considered | Intravenous N‑acetylcysteine shortens hospital length of stay compared with oral administration, resulting in lower total inpatient costs and overall lower costs. | No difference. |
| O’Brien, G.L. 2019[27] | Not considered | Subcutaneous trastuzumab substantially reduces direct medical and hospital overhead costs compared with intravenous administration, offering notable cost‑saving potential. It also saves considerable staff and patient time, improving the efficiency of hospital service delivery. | No difference. |
| Perraudin, C. 2020[29] | Not considered | The management costs of subcutaneous immunoglobulin therapy are significantly lower than those of intravenous immunoglobulin therapy. | No difference. |
| Rojas, L. 2020[30] | Not considered | Subcutaneous trastuzumab uses fewer consumables and less healthcare professional time than intravenous administration, leading to lower management costs. It also requires fewer vials and less patient time. | No difference. |
| Rønborg, S. 2016[31] | Assumption | Sublingual Dermatophagoides pteronyssinus tablets have higher drug acquisition costs than subcutaneous injections, but require fewer clinic visits, resulting in lower hospital costs, less patient time loss, and reduced travel and productivity costs. | No difference. |
| Simoens, S. 2021[34] | Not considered | Intravenous trastuzumab incurs higher healthcare professional time and consumable‑related costs than subcutaneous administration. | No difference. |
| Sullivan, S.D. 1996[36] | Not considered | Oral ganciclovir has lower nursing costs and a lower incidence of adverse events than intravenous ganciclovir, resulting in lower costs for managing adverse events. | No difference. |
| Vidal-Alaball 2006[37] | Assuming equal adherence in both groups | Oral vitamin B12 has slightly higher drug acquisition costs than intramuscular injection but requires less nursing time, resulting in lower overall costs. | No difference. |

# Reference

1. Abramson A, Halperin F, Kim J, Traverso G. Quantifying the Value of Orally Delivered Biologic Therapies: A Cost-Effectiveness Analysis of Oral Semaglutide. J Pharm Sci. 2019 Sept;108(9):3138–45.

2. Abushanab D, Rouf PA, Al Hail M, Kamal R, Viswanathan B, Parappil H, et al. Cost-effectiveness of Oral Versus Intravenous Ibuprofen Therapy in Preterm Infants With Patent Ductus Arteriosus in the Neonatal Intensive Care Setting: A Cohort-based Study. Clin Ther. 2021 Feb;43(2):336-348.e7.

3. Alsina L, Montoro JB, Moral PM, Neth O, Pica MO, Sánchez-Ramón S, et al. Cost-minimization analysis of immunoglobulin treatment of primary immunodeficiency diseases in Spain. Eur J Health Econ. 2022 Apr;23(3):551–8.

4. Altini M, Gentili N, Balzi W, Musuraca G, Maltoni R, Masini C, et al. The challenge of sustainability in healthcare systems: economic and organizational impact of subcutaneous formulations for rituximab and trastuzumab in onco-hematology. Expert Rev Pharmacoecon Outcomes Res. 2021 June;21(3):503–9.

5. Alonso Torres AM, Arévalo Bernabé AG, Becerril Ríos N, Hellín Gil MF, Martínez Sesmero JM, Meca Lallana V, et al. Cost-Analysis of Subcutaneous vs Intravenous Administration of Natalizumab Based on Patient Care Pathway in Multiple Sclerosis in Spain. Pharmacoecon Open. 2023 May;7(3):431–41.

6. Ascher-Svanum H, Furiak NM, Lawson AH, Klein TM, Smolen LJ, Conley RR, et al. Cost-effectiveness of several atypical antipsychotics in orally disintegrating tablets compared with standard oral tablets in the treatment of schizophrenia in the United States. Journal of Medical Economics. 2012 Jan;15(3):531–47.

7. Björstad Å, Cardell LO, Hahn-Pedersen J, Svärd M. A Cost-Minimisation Analysis Comparing Sublingual Immunotherapy to Subcutaneous Immunotherapy for the Treatment of House Dust Mite Allergy in a Swedish Setting. Clin Drug Investig. 2017 June;37(6):541–9.

8. Carter JA, Dammerman R, Frost M. Cost-effectiveness of subdermal implantable buprenorphine versus sublingual buprenorphine to treat opioid use disorder. Journal of Medical Economics. 2017 Aug 3;20(8):893–901.

9. Mylonas C, Kourlaba G, Fountzilas G, Skroumpelos A, Maniadakis N. Cost-Minimization Analysis of Trastuzumab Intravenous Versus Trastuzumab Subcutaneous for the Treatment of Patients With HER2+ Early Breast Cancer And Metastatic Breast Cancer in Greece. Value in Health. 2014 Nov 1;17(7):A640–1.

10. Davies L, Maynard A. An economic exploration of oral and intravenous ganciclovir in the induction and maintenance treatment of AIDS-related cytomegalovirus retinitis. Int J STD AIDS. 1996 Oct 1;7(6):415–21.

11. Ellis AK, Gagnon R, Hammerby E, Lau A. Sublingual immunotherapy tablet for the treatment of house dust mite allergic rhinitis in Canada: an alternative to minimize treatment costs? Allergy Asthma Clin Immunol. 2019 Dec;15(1):27.

12. Ellis AK, Gagnon R, Hammerby E, Shen J, Gosain S. Sublingual immunotherapy tablet: a cost-minimizing alternative in the treatment of tree pollen-induced seasonal allergic rhinitis in Canada. Allergy Asthma Clin Immunol. 2021 Dec;17(1):66.

13. Ellis AK, Mack DP, Gagnon R, Hammerby E, Gosain S. Minimization of ragweed allergy immunotherapy costs through use of the sublingual immunotherapy tablet in Canadian children with allergic rhinoconjunctivitis. Allergy Asthma Clin Immunol. 2023 Jan 18;19(1):7.

14. Fargier E, Ranchon F, Huot L, Guerre P, Safar V, Dony A, et al. SMABcare study: subcutaneous monoclonal antibody in cancer care: cost-consequence analysis of subcutaneous rituximab in patients with follicular lymphoma. Ann Hematol. 2018 Jan;97(1):123–31.

15. Guo S, Bozkaya D, Ward A, O’Brien JA, Ishak K, Bennett R, et al. Treating relapsing multiple sclerosis with subcutaneous versus intramuscular interferon-beta-1a: modelling the clinical and economic implications. Pharmacoeconomics. 2009;27(1):39–53.

16. Hu S, Yang H, Chen Z, Leng X, Li C, Qiao L, et al. Clinical Outcome and Cost-Effectiveness Analysis of CSII Versus MDI in Children and Adolescent With Type 1 Diabetes Mellitus in a Public Health Care System of China. Front Endocrinol. 2021 Mar 30;12:604028.

17. Raghu VK, Rudolph JA, Jalal HJ, Smith KJ. Microsimulation Model to Compare Enteral and Parenteral Iron Supplementation in Children With Intestinal Failure. J Parenter Enteral Nutr. 2021 May;45(4):810–7.

18. Lazzaro C, Lopiano L, Cocito D. Subcutaneous vs intravenous administration of immunoglobulin in chronic inflammatory demyelinating polyneuropathy: an Italian cost-minimization analysis. Neurol Sci. 2014 July;35(7):1023–34.

19. Lazzaro C, Van Steen C, Aptel F, Schweitzer C, Angelillo L. Cost-Utility Analysis of STN1013001, a Latanoprost Cationic Emulsion, versus Other Latanoprost Formulations (Latanoprost) in Open-Angle Glaucoma or Ocular Hypertension and Ocular Surface Disease in France. Cagini C, editor. Journal of Ophthalmology. 2022 Apr 29;2022:1–13.

20. Lazzaro C, van Steen C, Billeit S, Frauenknecht H, Kallen C, Pfennigsdorf S, et al. Cost-Utility Analysis of a Latanoprost Cationic Emulsion (STN1013001) versus Other Latanoprost in the Treatment of Open-Angle Glaucoma or Ocular Hypertension and Concomitant Ocular Surface Disease in Germany. Clin Ophthalmol. 2022;16:323–37.

21. Lazzaro C, Van Steen C, Ghirelli G, Sacchi M, Sisto D, Uva M, et al. A latanoprost cationic emulsion (STN1013001) vs. other latanoprost formulations (Latanoprost) in open angle glaucoma/ocular hypertension and ocular surface disease: an Italian cost-utility analysis. Expert Review of Pharmacoeconomics & Outcomes Research. 2023 Feb 7;23(2):251–65.

22. Lee VW, Cheng FW. Cost-minimisation analysis of intravenous versus subcutaneous trastuzumab regimen for breast cancer management in Hong Kong. Hong Kong Med J. 2023 Feb 3;29(1):16–21.

23. Lin Z, Xuan J. Cost-effectiveness of aripiprazole orally disintegrating tablets in the treatment of schizophrenia in China. Expert Review of Pharmacoeconomics & Outcomes Research. 2020 Sept 2;20(5):549–57.

24. Liu C, Wu Y, Wang F, Sun S, Wei J, Tao L. Cost-utility analysis for sublingual versus intravenous edaravone in the treatment of amyotrophic lateral sclerosis. Orphanet J Rare Dis. 2024 Oct 28;19(1):400.

25. Marchetti A, Rossiter R. Managing acute acetaminophen poisoning with oral versus intravenous N-acetylcysteine: a provider-perspective cost analysis. Journal of Medical Economics. 2009;12(4):384–91.

26. Martin A, Lavoie L, Goetghebeur M, Schellenberg R. Economic benefits of subcutaneous rapid push versus intravenous immunoglobulin infusion therapy in adult patients with primary immune deficiency. Transfusion Medicine. 2013 Feb;23(1):55–60.

27. O’Brien GL, O’Mahony C, Cooke K, Kinneally A, Sinnott SJ, Walshe V, et al. Cost Minimization Analysis of Intravenous or Subcutaneous Trastuzumab Treatment in Patients With HER2-Positive Breast Cancer in Ireland. Clinical Breast Cancer. 2019 June;19(3):e440–51.

28. O’Cathail SM, Shaboodien R, Mahmoud S, Carty K, O’Sullivan P, Blagden S, et al. Intravenous Versus Oral Dexamethasone Premedication in Preventing Paclitaxel Infusion Hypersensitivity Reactions in Gynecological Malignancies. Int J Gynecol Cancer. 2013 Sept;23(7):1318–25.

29. Perraudin C, Bourdin A, Vicino A, Kuntzer T, Bugnon O, Berger J. Home-based subcutaneous immunoglobulin for chronic inflammatory demyelinating polyneuropathy patients: A Swiss cost-minimization analysis. Moccia M, editor. PLoS ONE. 2020 Nov 25;15(11):e0242630.

30. Rojas L, Muñiz S, Medina L, Peña J, Acevedo F, Pinto MP, et al. Cost-minimization analysis of subcutaneous versus intravenous trastuzumab administration in Chilean patients with HER2-positive early breast cancer. Buyukhatipoglu H, editor. PLoS ONE. 2020 Feb 5;15(2):e0227961.

31. Rønborg S, Johnsen CR, Theilgaard S, Winther A, Hahn-Pedersen J, Andreasen JN, et al. Cost-minimization analysis of sublingual immunotherapy versus subcutaneous immunotherapy for house dust mite respiratory allergic disease in Denmark. Journal of Medical Economics. 2016 Aug 2;19(8):735–41.

32. Rudis MI, Touchette DR, Swadron SP, Chiu AP, Orlinsky M. Cost-effectiveness of oral phenytoin, intravenous phenytoin, and intravenous fosphenytoin in the emergency department. Ann Emerg Med. 2004 Mar;43(3):386–97.

33. Saha S, Raval D, Shah K, Saxena D. Cost-effectiveness analysis of parenteral iron therapy compared to oral iron supplements in managing iron deficiency anemia among pregnant women. Health Econ Rev. 2024 Jan 2;14(1):3.

34. Simoens S, Vulto AG, Dylst P. Simulating Costs of Intravenous Biosimilar Trastuzumab vs. Subcutaneous Reference Trastuzumab in Adjuvant HER2-Positive Breast Cancer: A Belgian Case Study. Pharmaceuticals. 2021 May 11;14(5):450.

35. Troy Somerville K. Cost advantages of oral drug therapy for managing cytomegalovirus disease. American Journal of Health-System Pharmacy. 2003 Dec 1;60(suppl_8):S9–12.

36. Sullivan SD, Mozaffari E, Johnson ES, Wolitz R, Follansbee SE. An economic evaluation of oral compared with intravenous ganciclovir for maintenance treatment of newly diagnosed cytomegalovirus retinitis in AIDS patients. Clin Ther. 1996;18(3):546–58.

37. Vidal-Alaball J, Butler CC, Potter CC. Comparing costs of intramuscular and oral vitamin B12 administration in primary care: a cost-minimization analysis. Eur J Gen Pract. 2006;12(4):169–73.

38. Wang GHM, Svensson M, Shao H, Vouri SM, Park H. Cost-effectiveness analysis of monthly, 3-monthly, and 6-monthly long-acting injectable and oral paliperidone in adults with schizophrenia. JMCP. 2023 Aug;29(8):884–95.
